# The alphavirus nonstructural protein 2 NTPase induces a host translational shut-off through phosphorylation of eEF2 via cAMP-PKA-eEF2K signaling

**DOI:** 10.1371/journal.ppat.1011179

**Published:** 2023-02-27

**Authors:** Emmely E. Treffers, Ali Tas, Florine E. M. Scholte, Arnoud H. de Ru, Eric J. Snijder, Peter A. van Veelen, Martijn J. van Hemert

**Affiliations:** 1 Molecular Virology Laboratory, Department of Medical Microbiology, Leiden University Medical Center, Leiden, The Netherlands; 2 Center for Proteomics & Metabolomics, Leiden University Medical Center, Leiden, The Netherlands; Purdue University, UNITED STATES

## Abstract

Chikungunya virus (CHIKV) is a reemerging alphavirus. Since 2005, it has infected millions of people during outbreaks in Africa, Asia, and South/Central America. CHIKV replication depends on host cell factors at many levels and is expected to have a profound effect on cellular physiology. To obtain more insight into host responses to infection, stable isotope labeling with amino acids in cell culture and liquid chromatography-tandem mass spectrometry were used to assess temporal changes in the cellular phosphoproteome during CHIKV infection. Among the ~3,000 unique phosphorylation sites analyzed, the largest change in phosphorylation status was measured on residue T56 of eukaryotic elongation factor 2 (eEF2), which showed a >50-fold increase at 8 and 12 h p.i. Infection with other alphaviruses (Semliki Forest, Sindbis and Venezuelan equine encephalitis virus (VEEV)) triggered a similarly strong eEF2 phosphorylation. Expression of a truncated form of CHIKV or VEEV nsP2, containing only the N-terminal and NTPase/helicase domains (nsP2-NTD-Hel), sufficed to induce eEF2 phosphorylation, which could be prevented by mutating key residues in the Walker A and B motifs of the NTPase domain. Alphavirus infection or expression of nsP2-NTD-Hel resulted in decreased cellular ATP levels and increased cAMP levels. This did not occur when catalytically inactive NTPase mutants were expressed. The wild-type nsP2-NTD-Hel inhibited cellular translation independent of the C-terminal nsP2 domain, which was previously implicated in directing the virus-induced host shut-off for Old World alphaviruses. We hypothesize that the alphavirus NTPase activates a cellular adenylyl cyclase resulting in increased cAMP levels, thus activating PKA and subsequently eukaryotic elongation factor 2 kinase. This in turn triggers eEF2 phosphorylation and translational inhibition. We conclude that the nsP2-driven increase of cAMP levels contributes to the alphavirus-induced shut-off of cellular protein synthesis that is shared between Old and New World alphaviruses. MS Data are available via ProteomeXchange with identifier PXD009381.

## Author summary

Chikungunya virus (CHIKV) is an important human pathogen from the alphavirus family that has caused outbreaks in Africa, Asia and the Americas. CHIKV replication strongly depends on resources of the host cell, which undergoes many changes during infection. Here, we have used phosphoproteomics to obtain more insight into such changes, which revealed that CHIKV infection induces phosphorylation of eukaryotic elongation factor 2 (eEF2). eEF2 regulates the elongation step of translation and its phosphorylation can strongly reduce protein synthesis. Previously, the nsP2 C-terminal domain was shown to reduce cellular transcription, which can prevent antiviral responses and promotes diversion of cellular resources to virus replication. Here, we describe that nsP2 also causes a shut-off of host-cell translation, which is mediated by the enzymatic activity of the nsP2 NTPase domain which acts through a previously unnoticed mechanism that involves cAMP, PKA and eEF2. Alphavirus infection or overexpression of nsP2 increased the cellular cAMP concentration, a second messenger that activates several signaling pathways, including the PKA and eEF2 kinases, of which the latter phosphorylates eEF2. Our observations highlight how alphaviruses modulate host cell metabolism on different levels and may account for poorly understood host shut-off effects observed in alphavirus-infected cells.

## Introduction

Chikungunya virus (CHIKV) is a re-emerging human pathogen that has affected the lives of millions over the past decades. CHIKV has a 12-kb RNA genome of positive polarity and belongs to the *Alphavirus* genus of the *Togaviridae* family [1]. It is an arthropod-borne virus that can be transmitted by mosquitos from the *Aedes* genus [2]. Clinical symptoms of CHIKV infection can include a high fever, rash and severe persisting polyarthralgia [3]. The virus was first discovered in 1952 in Tanzania [4] and for a long time caused limited outbreaks only. In 2005–2006, however, the virus reemerged in an epidemic form on the African east coast and several Indian ocean islands, infecting millions of people as it spread across the Asian continent [5]. In the autumn of 2013, the virus reached the Caribbean island of Saint-Martin, which was the start of a massive outbreak in Central and South America [6]. Within a year, more than 1 million suspected cases of CHIKV infection were reported [7]. In the past decade, hundreds of infected travelers have returned to non-endemic countries and as a result small locally-transmitted outbreaks have occurred in e.g. Italy, France, and the USA [8–12].

The CHIKV replicative cycle depends on a wide range of interactions with the host cell, while the infection also triggers a variety of antiviral and stress responses [13]. To gain more insight into the changes occurring in the host cell during the course of infection, we previously performed a temporal quantitative proteomics analysis of CHIKV-infected cells [14]. Surprisingly, that study revealed that overall the changes in protein abundance following infection were quite limited, and mainly concerned decreases. An important reason for the lack of upregulation of specific host protein levels during CHIKV infection is the host shut-off induced by alphaviruses, which renders the cell unable to respond to the infection at the level of protein synthesis (starting ~8 hours post infection (h p.i.) in the case of CHIKV) [15]. However, cellular responses to infection may also include modulation of the activity of proteins and other pathways, e.g. by changes in the localization and post-translational modifications (PTM) of proteins, such as phosphorylation and ubiquitination. These modifications generally occur on a different time scale and allow for much more rapid responses than those that depend on changes in protein abundance. Such PTM-based responses would not be readily detected by the more commonly used quantitative proteomics approaches that only analyze changes in protein abundance. The addition or removal of PTMs may alter the function, location, activity or interaction partners of a protein without affecting its abundance [16–18]. PTMs can quickly (de)activate signaling pathways, leading to major changes within the cell [19]. Considering the limited changes in protein abundance in CHIKV-infected cells that we observed previously, we hypothesized that the cell may mainly respond to infection at the level of PTMs. We, therefore, used CHIKV-infected cells to study changes in host protein phosphorylation, one of the most widely used mechanisms of regulating protein function in eukaryotes [18]. A quantitative phosphoproteomics approach using stable isotope labeling by amino acids in cell culture (SILAC) [20] was used and, to our knowledge, resulted in the first in-depth analysis of the temporal changes in host protein phosphorylation status during alphavirus infection. Our study revealed that CHIKV infection resulted in a strong increase in phosphorylation on a specific residue (T56) of eukaryotic elongation factor 2 (eEF2). Alphavirus nsP2 was found to be responsible for this induction of eEF2 phosphorylation, which depended on the protein’s NTPase activity to cause an increase in cellular cAMP levels that triggered the downstream phosphorylation of eEF2 by eEF2 kinase. We believe that this nsP2 NTPase-induced eEF2 phosphorylation constitutes an additional mechanism of the alphavirus host shut-off that remained elusive thus far.

## Results

**Fig 1:**
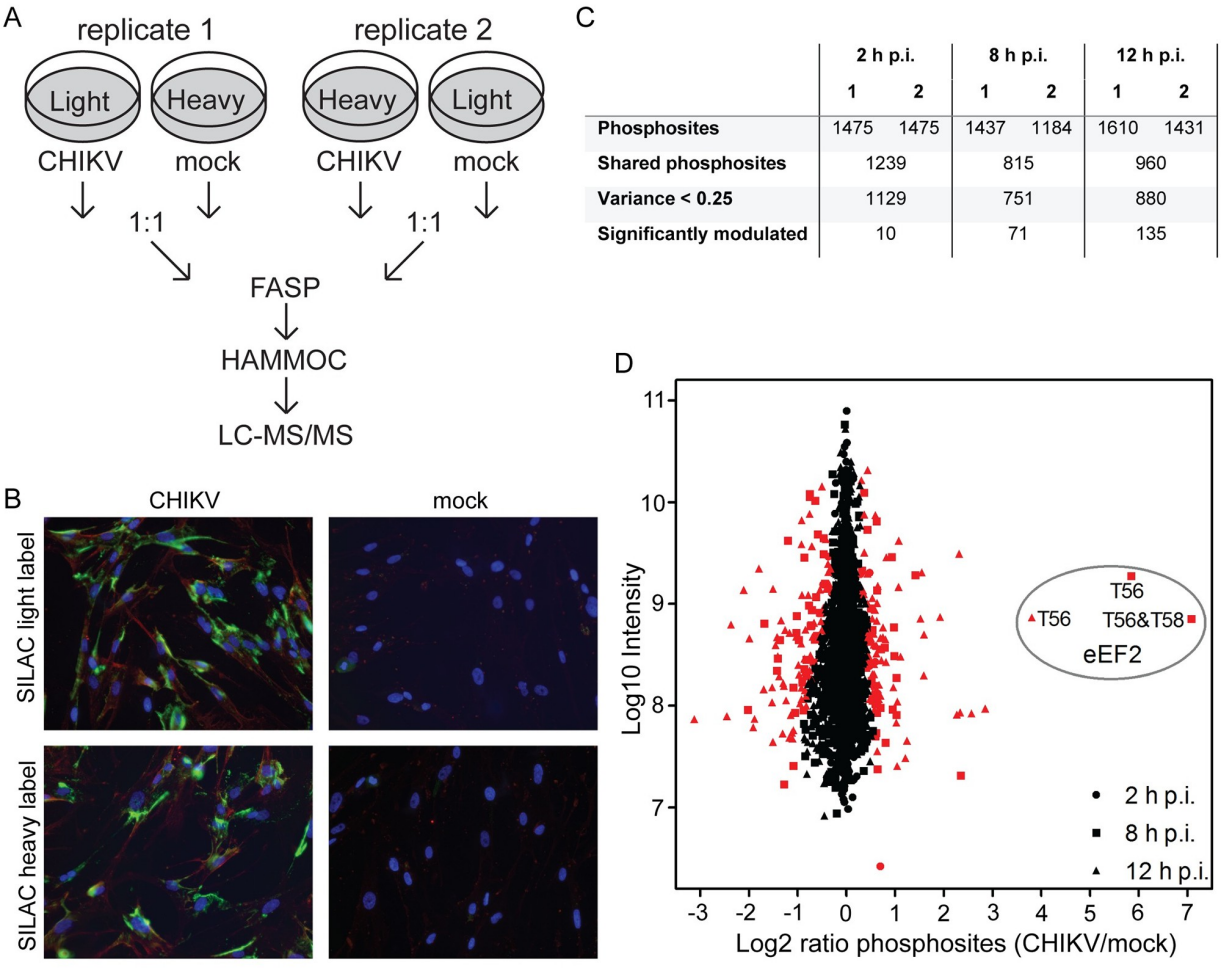
Phosphoproteomics analysis of CHIKV infection. (*A*) Experimental set-up of the SILAC-based phosphoproteomics study of virus-induced changes in the host proteome during the course of CHIKV infection. At each time point two biological replicates were used, in which a SILAC label-swap was performed between infected and mock-infected samples. Infected and mock-infected cells were lysed at 2, 8, and 12 h p.i. and equal amounts of protein were mixed and subsequently digested into peptides using the filter-aided sample preparation procedure. The samples were enriched for phosphopeptides using the HAMMOC procedure and analyzed by LC-MS/MS. (*B)* Immunofluorescence microscopy analysis of SILAC-labeled CHIKV-infected cells fixed at 12 h p.i. Cells were immunolabeled for dsRNA (green) and the CHIKV E2 protein (red), and nuclear DNA (blue) was stained with Hoechst-33342. (C) Number of identified phosphorylation sites at each time point p.i. The number of identified phosphorylation sites for each biological replicate and the total number of phosphorylation sites that were identified in both replicates at each time point p.i. are shown. For follow-up analysis only phosphorylation sites with a variance <0.25 were selected. (*D*) Proteome-wide quantification of phosphorylation sites at 2 (dots), 8 (squares), and 12 (triangles) h post-CHIKV infection. Average normalized log_2_ ratios (Infected/mock) are plotted against the log_10_ of peptide intensities measured in the mass spectrometer. Each mark represents a protein group (proteins (often isoforms) that are grouped together due to shared peptide identifications). Red marks indicate phosphorylation sites that were significantly modulated (Benjamini-Hochberg FDR of 0.05). The phosphorylation sites identified for eEF2 at 8 and 12 h p.i. are indicated.

To identify proteins of which the phosphorylation status changes upon CHIKV infection, we performed a SILAC time-course experiment and compared the protein composition of lysates of CHIKV- and mock-infected MRC-5 cells (human lung fibroblasts). Samples harvested at 2, 8, and 12 h p.i. were enriched for phosphopeptides, followed by LC-MS/MS analysis and phosphorylation site quantification (Fig 1A). Successful CHIKV infection of SILAC-labeled cells was confirmed by immunofluorescence microscopy, and by 12 h p.i. practically all cells stained positive for both double-stranded (ds) RNA (a marker for viral replication) and the CHIKV E2 protein (Fig 1B).

**Fig 2:**
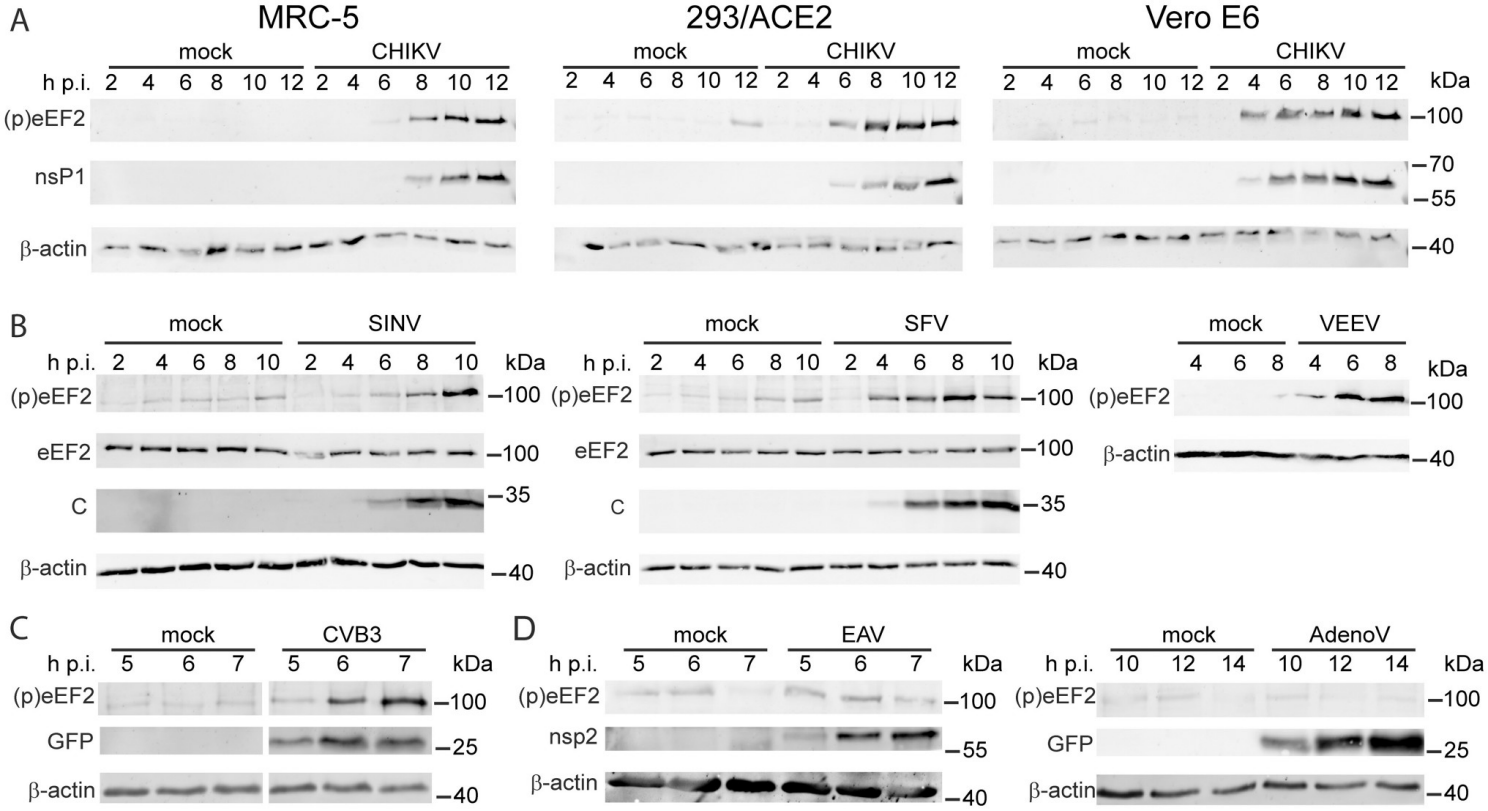
Alphavirus and enterovirus infection triggers early phosphorylation of eEF2 on T56. (*A*) eEF2 phosphorylation is triggered in CHIKV-infected MRC-5, 293/ACE2 and Vero E6 cells (MOI 5) at different time points post infection. Viral protein nsP1 and (p)eEF2 (T56) were detected by WB. (*B*) eEF2 phosphorylation is triggered in SINV- and SFV-infected Vero E6 cells at different time points post infection (MOI 5). Viral protein C, eEF2 and (p)eEF2 (T56) were detected by WB. (*C*) eEF2 phosphorylation is triggered in CVB3-GFP infected VeroE6 cells at different times post infection (MOI 1). GFP expression was used to monitor progression of the infection and (p)eEF2 (T56) was detected by WB analysis. (*D*) eEF2 phosphorylation is not triggered in EAV infected BHK-21 cells (MOI 5) or AdenoV-GFP infected Vero E6 cells. Viral protein nsp2 or GFP, and (p)eEF2 (T56) were detected by WB. Western blots are representative of at least two independent replicates.

Our earlier study [14] demonstrated that until 8 h p.i. changes in the host proteome at the level of protein abundance were minimal, but we expected changes in phosphorylation status to occur more rapidly and, therefore, decided to also analyze samples taken at 2 h p.i. and monitor the earliest responses to CHIKV infection. By 8 h p.i., the viral proteins were clearly detectable by Western blot (WB) analysis in this cell line (Fig 2A) and by 12 h p.i. high levels of viral protein and RNA were present but cytopathic effects were not yet observed [21].

### Changes in the phosphoproteome during CHIKV infection

Per time point, ~800–1200 phosphorylated peptides were quantified in both biological replicates. (Fig 1C and 1D). In total 2988 phosphorylation sites were identified on 1060 proteins (S1 Table). Phosphorylated peptides derived from CHIKV proteins were also identified. Two phosphorylation sites were identified on the capsid protein (C), two on envelope protein E2, and four on nonstructural protein 3 (nsP3). (S1 Table)

In total, 167 unique phosphorylation sites on 113 host proteins were modulated significantly with a Benjamini-Hochberg false discovery rate (FDR) of 0.05. Several of these sites were significantly modulated at more than one time point (Fig 1D and S2 Table). The Maximal Motif Finder for Phosphoproteomics datasets was used to find overrepresented motifs [22]. Three motifs were identified, TP (13 sites), SP (75 sites) and RxxS (34 sites).

At 2 h p.i., the overall changes in phosphorylation status were minimal, although 10 sites were significantly modulated, with four of these also being significantly modulated at the two later time points: T56 on eEF2 (Log_2_ ratio 0.70), S430 on vimentin (Log_2_ ratio 0.48), and T185 and Y187 on mitogen-activated protein kinase (MAPK) (Log_2_ ratio -0.48 and 0.29, respectively). At 8 h p.i., the phosphorylation status of 71 sites had changed significantly and by 12 h p.i. this number had increased to 136. Of these, 42 sites were significantly modulated at both 8 and 12 h p.i. (S2 Table). Both increases (110 occurrences) and decreases (107 occurrences) in phosphorylation were observed. On 11 peptides we identified sites with a larger than 4-fold change in phosphorylation status on at least one of the time points studied (Table 1).

**Table 1:** Peptides that contain a phosphorylation site that changed phosphorylation status >4 fold on at least one of the time points analyzed.

Protein	Accession number	Peptide sequence	Modified residue(s) #	Log2 ratio		
				2 h p.i.	8 h p.i.	12 h p.i.
EEF2	P13639	AGETRFT(ph)DTR	T56	0.70 *	5.85 *	3.81 *
		AGETRFT(ph)DT(ph)RKDEQER	T56, T58	NaN	7.08 *	NaN
HSPB1	P04792	ALS(ph)RQLS(ph)SGVSEIR	S78, S82	0.05	0.35	2.31 *
LARP1	Q6PKG0	S(ph)LPTTVPES(ph)PNYR	S769, S774	-0.02	-1.37 *	-2.11 *
MTDH	E5RJU9	SETSWES(ph)PK	S568	-0.06	-2.02 *	-3.12 *
NES	P48681	S(ph)LGEEIQESLK	S548	NaN	1.03 *	2.27 *
		SLRS(ph)LEEQDQETLR	S746	NaN	NaN	2.85 *
NUP50	Q9UKX7-2	VAAETQS(ph)PSLFGSTK	S221	NaN	1.30	2.34 *
PRRC2C	Q9Y520-2	AFGSGIDIKPGT(ph)PPIAGR	T2673	-0.14	-1.62	-2.45 *
RALY	Q9UKM9-2	TRDDGDEEGLLTHS(ph)EEELEHS(ph)QDTDA DDGALQ	S288, S295	-0.27	-2.17	-2.37 *
SH3KBP1	B7Z6E8	ANS(ph)PSLFGTEGKPK	S587	NaN	2.35 *	2.57 *

(ph) indicates the preceding amino acid residue was modified by phosphorylation.

^#^ amino acid residue number according to PhosphoSitePlus [23].

*Significantly changed phosphorylation status at this time point with Benjamini-Hochberg FDR 0.05. NaN: peptide was not identified at this time point.

### CHIKV infection triggers an early and strong phosphorylation of eEF2

The protein that showed the largest increase in phosphorylation, i.e. a >50 fold increase at 8 and 12 h p.i., was eEF2, which became phosphorylated on T56 and T58 (Fig 1D). eEF2 is a GTPase that is required for the translocation step of the nascent peptide chain during translation elongation [24]. The eEF2 kinase (eEF2K) regulates the translation elongation rate by inactivating eEF2 through sequential phosphorylation of T56 and T58 [25], but phosphorylation on T56 alone suffices to inactivate eEF2 as it reduces the protein’s affinity for the ribosome through a conformational change [26,27]. In concurrence with this key role of T56, we only identified phosphorylated T58 on a peptide also containing phosphorylated T56 (Table 1). Phosphorylation of eEF2 can be reversed by protein phosphatase 2A (PP2A) when more active eEF2 is required for translation [28].

Because of the very strong increase in phosphorylation, eEF2 was selected for follow-up analysis and its phosphorylation on T56 was confirmed by WB using samples from independent time-course experiments in CHIKV-infected MRC-5, 293/ACE2, and Vero E6 cells (Fig 2A). In the proteomics experiments, a significant increase in phosphorylation (Log_2_ ratio 0.70) was already observed at 2 h p.i. Likewise, in Vero E6 cells, strong phosphorylation on this residue could be detected early in infection (4 h p.i.) by WB analysis (Fig 2A). The sensitivity of WB is, however, much lower compared to the MS-based analysis, which explains why phosphorylation of eEF2 in MRC-5 cells only became visible in WB from 6 h p.i onwards.

### Several other viruses also trigger phosphorylation of eEF2

We next explored whether eEF2 phosphorylation also occurs upon infection with other viruses. The alphaviruses are subdivided into Old World and New World alphaviruses, which evolved separately [29]. CHIKV belongs to the Old World alphaviruses. In Vero E6 cells infected with two other Old World alphaviruses, Sindbis virus (SINV) and Semliki Forest virus (SFV), increased eEF2 phosphorylation was detected 8 and 4 h p.i. respectively. In Vero E6 cells infected with the New World alphavirus Venezuelan equine encephalitis virus (VEEV) increased eEF2 phosphorylation was detected 4 h p.i. (Fig 2B), suggesting that this modification is common in alphavirus-infected cells. We subsequently tested three unrelated viruses, a GFP-expressing recombinant coxsackie B3 virus (CVB3) (+RNA virus, picornavirus family), equine arteritis virus (EAV) (+RNA virus, arterivirus family, order *Nidovirales*), and a GFP-expressing human adenovirus type 5 (HAdV) (dsDNA virus, adenovirus family). Infection with CVB3 resulted in early eEF2 phosphorylation (Fig 2C), but infections with EAV or HAdV did not (Fig 2D).

### eEF2 phosphorylation is not triggered by viral (ds)RNA

**Fig 3:**
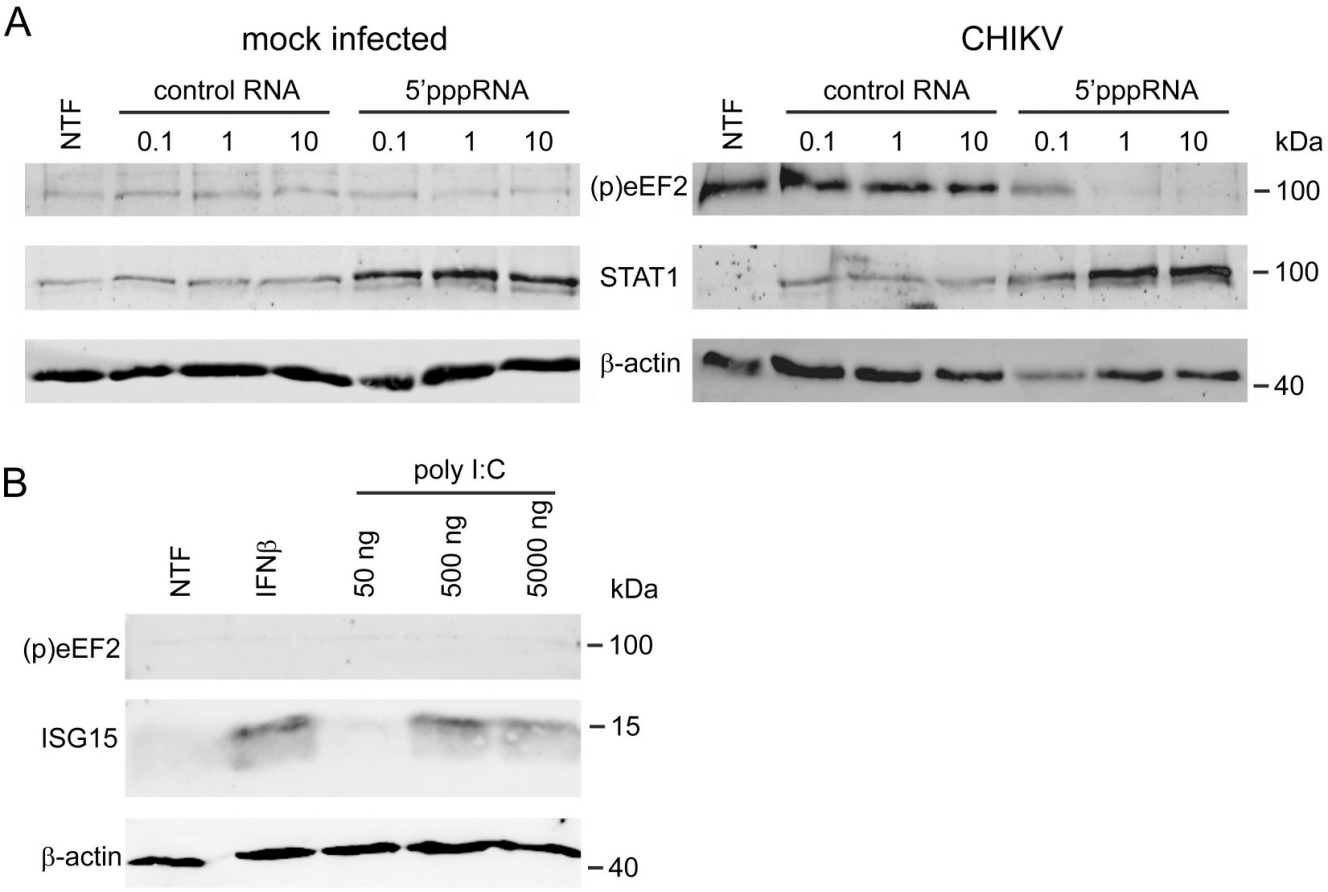
RIG-I & MDA5 activation does not trigger eEF2 phosphorylation. (*A*) MRC-5 cells were transfected with 0.1, 1, or 10 ng/ml 5’pppRNA or a control RNA 1 hour prior to infection with CHIKV-LS3-GFP (MOI 0.1) and non-transfected cells (NTF) were included as control. Protein lysates were harvested 24 h p.i. and STAT1 and (p)eEF2 (T56) were detected by WB analysis. (*B*) MRC-5 cells were transfected with 50, 500 or 5000 ng/ml poly I:C or treated with 200 U/ml IFNβ and harvested at 24 h p.t. (p)eEF2 (T56) and ISG15 were detected by WB. Western blots are representative of two independent replicates.

Since eEF2 phosphorylation was induced by virus infection, we hypothesized that it might be part of an innate immune response that is triggered by the sensing of certain pathogen-associated molecular patterns (PAMPs), e.g. viral dsRNA replication intermediates, via the RIG-I/MDA5-MAVS pathways. To test this possibility, RIG-I was activated by transfection of MRC-5 cells with non-cytotoxic doses of 5’-triphoshorylated RNAs (5’pppRNA) representing sequences from the 5’- and 3’-untranslated regions of the vesicular stomatitis virus genome. Previously, this treatment was shown to induce a robust antiviral response to CHIKV infection as it reduced the intracellular viral RNA, viral protein and viral titer in a dose-dependent manner [30]. However, this RIG-I agonist did not induce eEF2 phosphorylation, despite the fact that an innate immune response was triggered as indicated by the induction of STAT1 expression (Fig 3A). In CHIKV-infected cells, 5’pppRNA transfection prevented eEF2 phosphorylation in a dose-dependent manner, likely because overall CHIKV replication was inhibited by the 5’pppRNA treatment (Fig 3A). MDA5 was activated by transfection of MRC-5 cells with a non-cytotoxic dose of the dsRNA analog poly I:C. Also this treatment did not induce eEF2 phosphorylation, while ISG15 expression was induced as expected [31] (Fig 3B). Treatment of MRC-5 cells with interferon-β (IFNβ) also resulted in the induction of ISG15 expression, while no eEF2 phosphorylation was observed (Fig 3B). These findings indicate that it is unlikely that eEF2 phosphorylation is part of an innate immune response to the production of viral (ds)RNA.

### Viral structural proteins and virus entry are not required for induction of eEF2 phosphorylation

**Fig 4:**
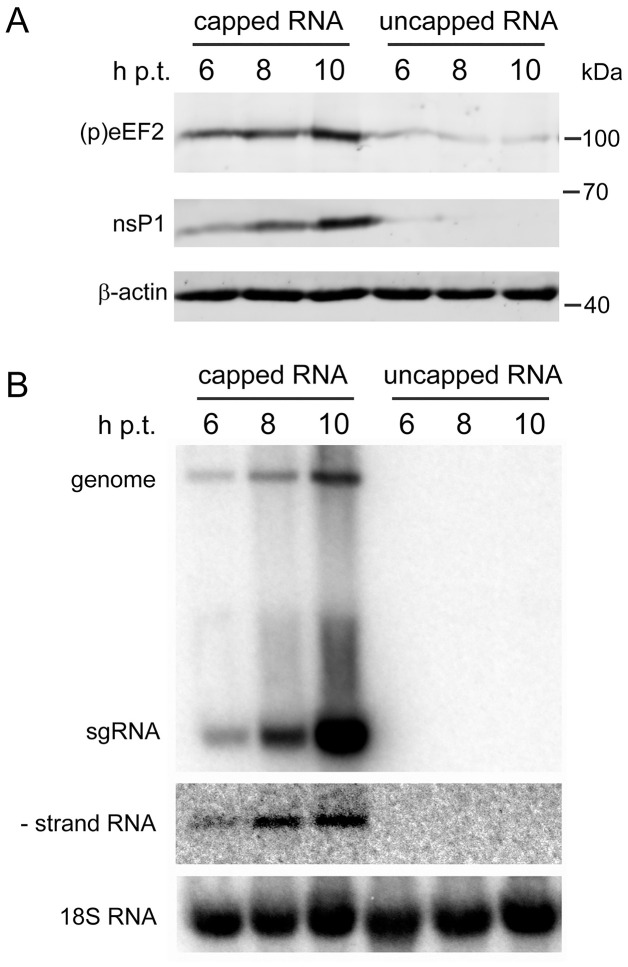
Transfection of a capped CHIKV replicon triggers eEF2 phosphorylation. BHK-21 cells were electroporated with *in vitro* transcribed capped or uncapped CHIKV LS3 replicon RNA. Protein lysates were harvested and RNA was isolated at 6, 8 and 10 h p.i. (*A*) Viral protein nsP1 and (p)eEF2 (T56) were detected by WB. (*B*) Positive- and negative strands of CHIKV RNA were detected by in-gel hybridization with radioactively labeled oligonucleotides complementary to the 3’ end of either negative- or positive-strand CHIKV RNA. The cellular 18S ribosomal RNA was used as a loading control. Figures are representative of two independent replicates.

To determine whether the CHIKV structural proteins play a role in triggering eEF2 phosphorylation, BHK-21 cells were transfected with a CHIKV RNA replicon that does not express any of the structural proteins and eEF2 phosphorylation was monitored by WB. As transfection of this replicon induced a strong eEF2 phosphorylation (Fig 4A), we concluded that none of the structural proteins is responsible for the induction. As a control, we included an uncapped version of the replicon RNA transcript, which cannot be translated and was found not to induce eEF2 phosphorylation. An analysis of intracellular RNA revealed a steady increase of the levels of genome, subgenomic mRNA (sgRNA) and negative-stranded RNA in cells transfected with the capped replicon, but the uncapped RNA was most likely quickly degraded as it could not be detected at 6 hours post transfection (h p.t.) (Fig 4B). Since regular endocytosis-mediated viral entry was bypassed in these transfection experiments, we concluded that eEF2 phosphorylation does not depend on the viral entry process.

### The helicase-associated NTPase domain of nsP2 triggers eEF2 phosphorylation

**Fig 5:**
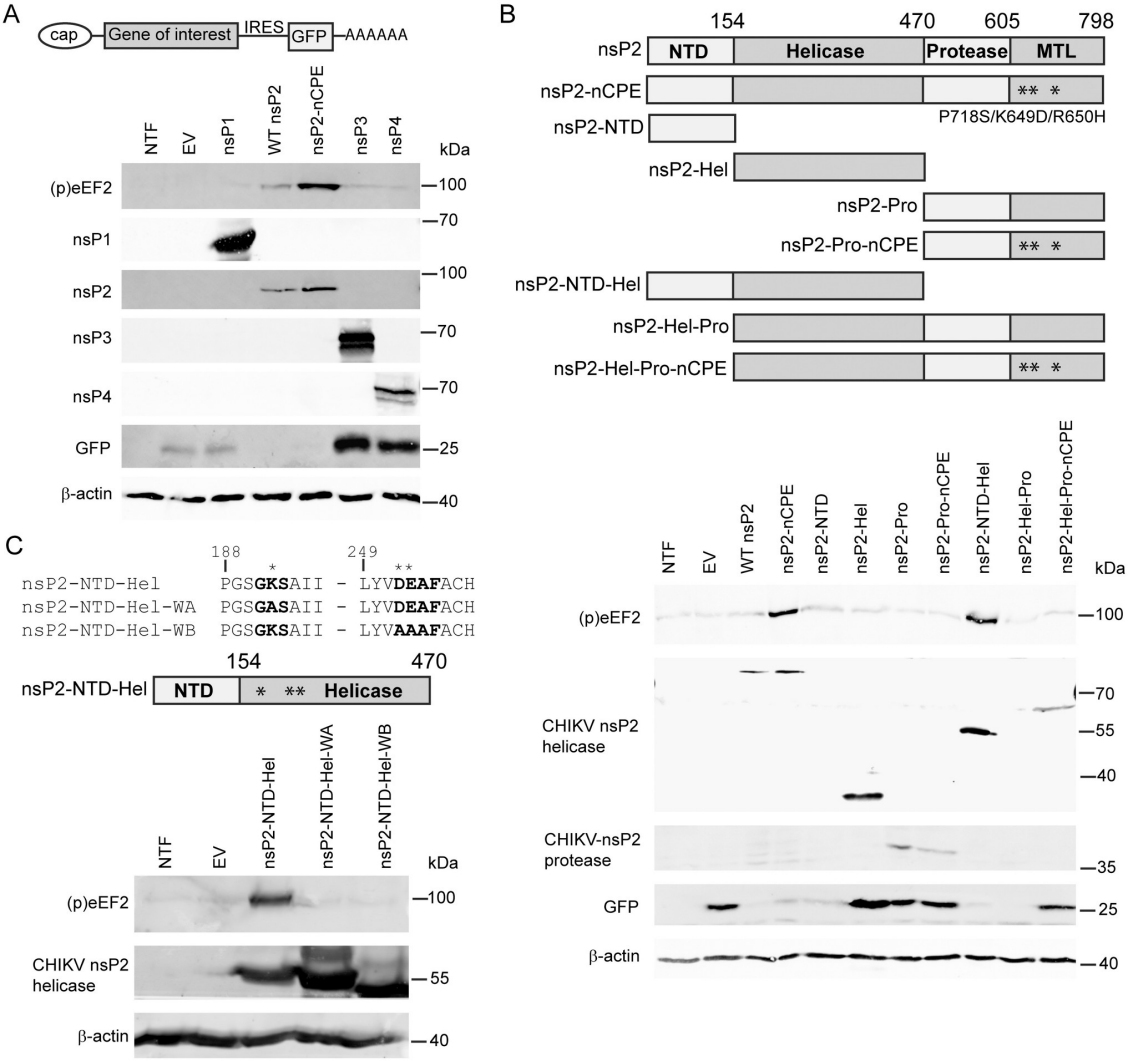
The helicase-associated NTPase domain of nsP2 triggers eEF2 phosphorylation. (*A*) 293T cells were transfected with the pCAGGS-IRES-GFP construct containing individual CHIKV nsPs. (p)eEF2 (T56), GFP and viral proteins, nsP1, nsP2, nsP3 and nsP4 were detected by WB. (*B*) 293T cells were transfected with the pCAGGS-IRES-GFP construct containing full-length CHIKV nsP2 or different truncations. Top: schematic view of the truncations that were used. NTD: N-terminal domain, MTL: methyl-transferase-like. Amino acid (AA) numbers are indicated in figure. * indicates mutations in the nCPE constructs. Bottom: (p)eEF2 (T56), GFP and CHIKV nsP2 helicase, CHIKV nsP2 protease and GFP were detected by WB. (*C*) 293T cells were transfected with the pCAGGS-IRES-GFP construct containing the nsP2-NTD-Hel WT sequence or 2 Walker motif mutants. Top: AA sequence surrounding Walker A (WA) and Walker B (WB) motifs (indicated in bold) and schematic view of CHIKV nsp2-NTD-Hel construct that was used. * indicates residues that were mutated in the 2 mutants. Bottom: (p)eEF2 (T56) and CHIKV nsP2 helicase were detected by WB. For all panels cells transfected with an empty vector and non-transfected cells (NTF) were included as controls. Representative blots are shown and each experiment was repeated at least 3 times.

Having established that the CHIKV structural proteins are not required for induction of eEF2 phosphorylation, we surmised that the nonstructural polyprotein or one of its cleavage products could be responsible. To test whether any of the four CHIKV nsPs triggered eEF2 phosphorylation, their coding sequences were individually cloned into a modified pCAGGS vector (pCAGGS-IRES-GFP). This vector also expresses GFP from an encephalomyocarditis virus internal ribosomal entry site (IRES) positioned downstream of the gene of interest (Fig 5A). Plasmids were transfected into 293T cells and eEF2 phosphorylation and expression of the individual CHIKV nsPs was monitored by WB. IRES-driven expression of the GFP marker was observed in cells transfected with all expression constructs (empty vector (EV) and nsP1, nsP3, and nsP4) except for the nsP2 expression construct, for reasons explained below (Fig 5A).

Expression of nsP1, nsP3, or nsP4 did not induce eEF2 phosphorylation, but expression of nsP2 did (Fig 5A). Expression of wild-type (WT) CHIKV nsP2 was, however, toxic to cells and in this manner inhibited its own expression, as well as that of the IRES-driven GFP reporter gene encoded in the same construct. This was due to the known nsP2-mediated host shut-off [13], to which eEF2 phosphorylation likely also contributes.

To circumvent this problem and boost nsP2 expression, we next employed a plasmid expressing a non-cytopathic mutant of nsP2 (nCPE P718S/K649D/R650H), which contained three previously described mutations that prevent host shut-off [32,33]. As shown in Fig 5A, nsP2-nCPE expression induced eEF2 phosphorylation to a much higher level than in cells expressing WT nsP2, but GFP expression was still inhibited.

We hypothesized that one of nsP2’s domains or activities may trigger a pathway that induces eEF2 phosphorylation during infection. The protein is responsible for the host transcriptional shut off [34–39]. The N-terminal part of nsP2 contains an N-terminal domain (NTD) with unknown function and the viral RNA helicase and its associated RNA triphosphatase (RTPase)/nucleoside triphosphatase (NTPase) [40–42]. The C-terminal part of nsP2 contains the protease domain that mediates cleavage of the nonstructural polyprotein into the individual nsPs [43,44] and also a methyl transferase-like (MTL) domain, but this is presumed to be enzymatically inactive [45]. To delineate the part of nsP2 relevant for eEF2 phosphorylation, its N-terminal (NTD, aa 1–154), helicase (Hel, aa 155–470) and protease (Pro, aa 471–798) domains were expressed using pCAGGS-IRES-GFP, both as individual domains and in combination with adjacent domains (Fig 5B). For constructs containing the Pro domain, also variants with the nCPE mutations were constructed to boost expression of these proteins. Plasmids were transfected into 293T cells and eEF2 phosphorylation was monitored by WB. Expression of nsP2-NTD-Hel strongly induced eEF2 phosphorylation, while expression of the individual NTD and Hel domains did not. The Pro domain was not required for the induction of eEF2 phosphorylation. Successful expression of all protein products was confirmed by WB, with the exception of nsP2-NTD, due to the lack of a suitable antibody.

The CHIKV nsP2 helicase is a member of helicase superfamily 1 and includes two RecA-like domains containing multiple conserved sequence motifs. [46–48]. However, the nsP2-NTD-Hel protein expressed from our construct is not expected to be a functional RNA helicase, as in vitro enzymatic assays demonstrated previously that sequences from the C-terminal part of nsP2 are indispensable for helicase activity [36,49]. This truncated form of nsP2 is, however, expected to still exhibit NTPase and RTPase activity, which can be inactivated or reduced by mutating key residues of its conserved Walker A and B (WA and WB) motifs [49,50], which are critical for NTP binding. Mutations specifying amino acid substitutions in either the WA (GKS -> GAS, aa 192) or the WB (DEAF -> AAAF, aa 252 and 253) motif were introduced into pCAGGS-IRES-GFP-nsP2-NTD-Hel. Both mutant proteins were unable to induce any eEF2 phosphorylation, while we noticed that their expression level was substantially higher than that of WT nsP2-NTD-Hel (Fig 5C). The WB mutant protein migrated slightly faster in SDS-PAGE, most likely due to its double charged-to-alanine substitution. Taken together, the above observations directly implicated the NTPase activity of CHIKV nsP2 in the induction of eEF2 phosphorylation, in expression systems and–most likely–also in infected cells.

### Decreased ATP and increased cAMP levels during alphavirus infection are associated with eEF2 phosphorylation

**Fig 6:**
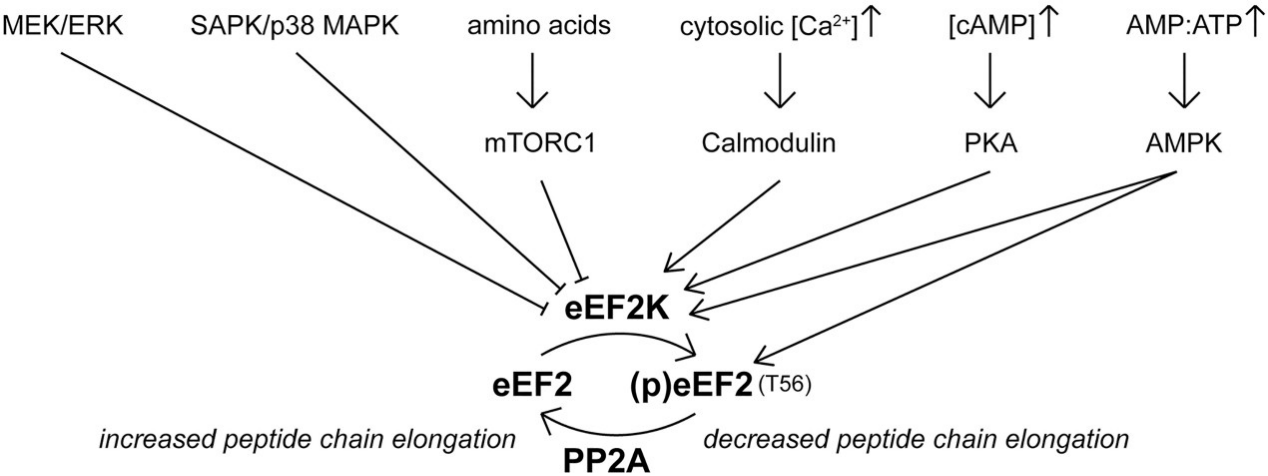
Regulation of eEF2 phosphorylation. Schematic overview of different pathways involved in regulating eEF2 phosphorylation. An increase in the cytosolic Ca^2+^ concentration activates eEF2K. Activation of PKA by an increased concentration of cAMP activates eEF2K. Activation of AMPK by an increase in the AMP:ATP ratio activates eEF2K and AMPK possibly also directly phosphorylates eEF2. eEF2K can be inhibited by mTORC1, the MEK/ERK pathway and SAPK/p38 MAP kinases. eEF2 T56 phosphorylation is reversible through dephosphorylation by PP2A.

**Fig 7:**
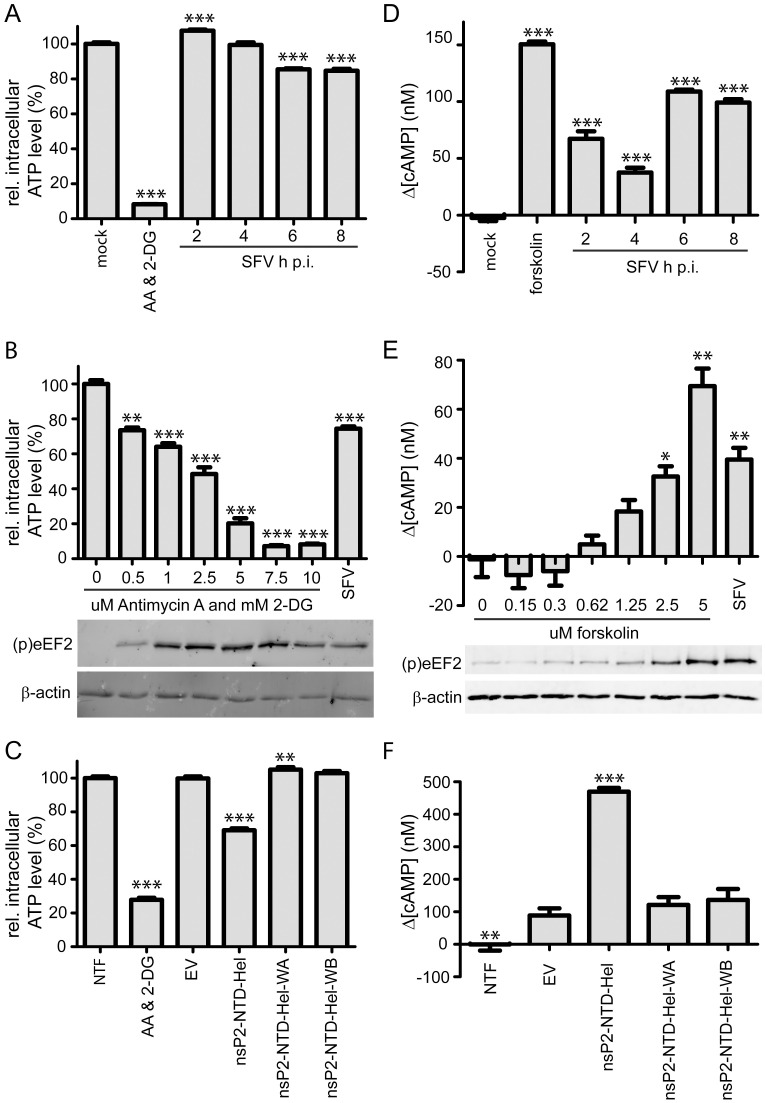
Changes in cellular ATP and cAMP levels are associated with CHIKV nsP2 induced eEF2 phosphorylation. For panels A, B and C the CellTiter-Glo 2.0 assay was used to determine relative cellular ATP levels and for panels D, E and F the cAMP-Glo assay was used to determine changes in cellular cAMP concentrations. (*A*) Vero E6 cells were infected with SFV (MOI 10) and at 2, 4, 6 and 8 h p.i. cellular ATP levels were determined relative to the level in mock-infected cells at 8 h p.i. A combination of ATP-depleting compounds antimycin A (AA) (10 μM) and 2-DG (10 mM) was used as a control. Bars represent the averages of two independent experiments with 8 replicates each. (*B*) Vero E6 cells were incubated for 30 min with increasing concentrations of antimycin A and 2-DG. (p)eEF2 (T56) was detected by WB. Cells infected with SFV (MOI 10) at 6 h p.i. were included as a positive control. Bars represent the averages of two independent experiments with two or three replicates each. P-value ** <0.01, *** <0.0001 compared to no compounds added (*C*) 293T cells were transfected with the pCAGGS-IRES-GFP construct containing the nsP2-NTD-Hel WT sequence or 2 Walker motif mutants. Cells transfected with an empty vector and non-transfected cells (NTF) were included as negative controls. Cells treated with 10 μM antimycin A (AA) and 10 mM 2-DG for 30 min were included as positive control. Bars represent the average of two independent experiments with 8 replicates each. P-value ** <0.01, *** <0.0001 compared to EV control. (*D*) Vero E6 cells were infected with SFV (MOI 10) and at 2, 4, 6 and 8 h p. i. changes in cAMP concentration were determined relative to the level in mock infected cells at 8 h p.i. 5 μM of cAMP increasing compound forskolin was used as a positive control. *** p-value <0.0001 compared to mock. (*E*) Vero E6 cells were incubated for 30 min with increasing concentrations of forskolin. (p)eEF2 (T56) was detected by WB. Cells infected with SFV (MOI 10) at 6 h p.i. were included as a positive control. P-value * <0.05, ** <0.01, *** <0.0001 compared to no forskolin added. (*F*) 293T cells were transfected with the pCAGGS-IRES-GFP construct containing the nsP2-NTD-Hel WT sequence or 2 Walker motif mutants. Cells transfected with an empty vector and non-transfected cells (NTF) were included as negative controls. Bars are of representative example of experiment with 8 replicates, experiment was repeated twice. Statistical differences were determined by comparing with the NTF control, p-value ** <0.01, *** <0.0001.

The activity of the kinase controlling eEF2 phosphorylation (eEF2K) is regulated by a variety of signaling pathways, including the Ca^2+/^calmodulin, PKA, AMPK, mTORC1 and MAPK pathways (Fig 6) [30]. Knowing that the NTPase activity of nsP2 is involved in eEF2 phosphorylation, we hypothesized that the direct trigger could be a reduction in ATP levels that would increase the AMP concentration and the AMP:ATP ratio, which would in turn activate the 5’AMP activated protein kinase (AMPK). Alternatively, the protein kinase A (PKA) pathway might be responsible for eEF2K activation in response to an increased intracellular cAMP concentration (Fig 6) [51]. We, therefore, monitored the changes in cellular ATP and cAMP levels at different time points post infection. As the plate reader required for the CellTiter-Glo 2.0 and the cAMP-Glo assays that were used to determine ATP and cAMP levels was not available in our BSL-3 facility, they had to be performed using SFV-, rather than CHIKV-infected cells, as the former can be used in a BSL-2 environment. Following SFV infection, ATP levels dropped to about 85% of those in the mock-infected control by 6 h p.i. (Fig 7A). Intracellular cAMP levels had increased by ~65 and ~100 nM by 2 and 6 h p.i., respectively (Fig 7D).

To check whether the observed drop in ATP concentration was sufficient to induce eEF2 phosphorylation, different concentrations of the ATP-depleting compounds antimycin A and 2-deoxyglucose (2-DG) [52] were used to simulate the situation during viral infection. In a direct comparison, WB analysis showed that the observed drop in ATP levels during SFV infection also induced eEF2 phosphorylation when it was mimicked by a combined treatment with 0.5 uM antimycin A and 0.5 mM 2-DG (Fig 7B). To check whether the observed increase in cAMP level was sufficient to induce eEF2 phosphorylation, different concentrations of the cAMP inducing compound forskolin [53] were used. WB analysis showed that the increase in cAMP concentration observed during SFV infection also induced eEF2 phosphorylation when it was mimicked with a 2.5 uM forskolin treatment (Fig 7E).

Overexpression of nsP2-NTD-Hel resulted in a ~30% decrease in ATP level and a more than 350 nM increase in cAMP concentration compared to the EV control, but when the WA and WB mutants were overexpressed the ATP and cAMP levels remained unchanged, highlighting the importance of a catalytically active NTPase domain (Fig 7C and 7F). These observations strongly suggest that the changes in intracellular ATP and cAMP levels are directly linked to the NTPase activity of nsP2, leading to changes that ultimately trigger the activation of eEF2K and subsequent eEF2 phosphorylation. Since a rise in cAMP concentration could already be observed at 2 h p.i., which is consistent with the significant increase in eEF2 phosphorylation at this same time point in the proteomics experiment (Table 1), cAMP likely is the main trigger for eEF2 phosphorylation during alphavirus infection.

### CHIKV nsP2-induced eEF2 phosphorylation occurs independent of AMPK

**Fig 8:**
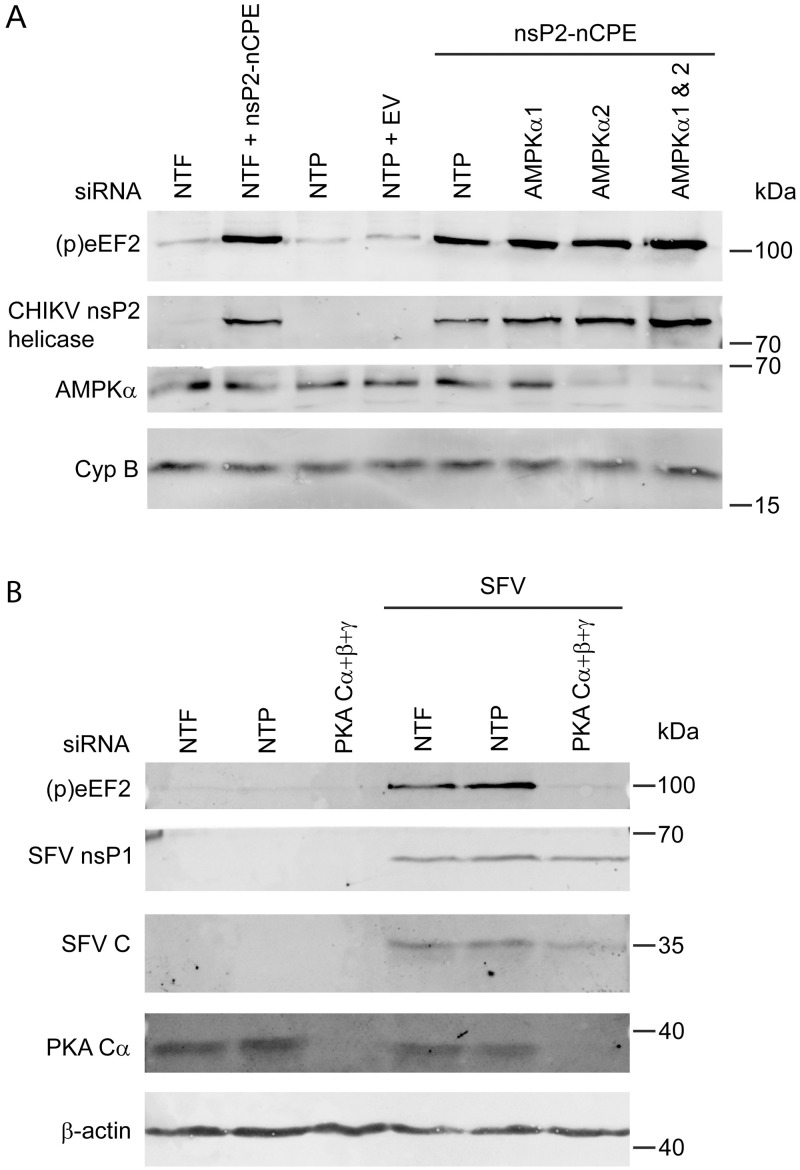
CHIKV nsP2-induced phosphorylation occurs independent of AMPK. *(A)* Vero E6 cells were transfected with siRNAs targeting the catalytic subunit isoforms of AMPK. A non-targeting pool (NTP) of siRNAs was used as a control. 2 d p.t. the cells were transfected with pCAGGS-IRES-nsP2-nCPE and harvested 18 h p.t. An empty vector transfection and non-transfected cells (NTF) were included as controls. nsP2, (p)eEF2 (T56), and AMPKα were detected by WB. A representative blot is shown of 3 independent replicates. *(B)* Vero E6 cells were transfected with siRNAs targeting the catalytic subunits of PKA. A non-targeting pool (NTP) of siRNAs and non-transfected cells (NTP) were used as a control. 3 d p.t. the cells were infected with SFV MOI 1 and harvested 6 h p.i. (p)eEF2, nsP1, PKA and C were detected by WB.

Since eEF2 phosphorylation appeared to be induced upon a drop in ATP and/or increase in cAMP levels, we focused our studies on the AMPK and PKA pathways that can activate eEF2K in response to changes in the AMP:ATP ratio and cAMP concentration, respectively [51]. AMPK is a heterotrimeric holoenzyme consisting of a catalytic α subunit and regulatory β and γ subunits. There are two isoforms of the catalytic subunit (α1 and α2) and expression is cell type dependent [54]. AMPK was previously described as an antiviral sensor [55] and our phosphoproteomics experiment indicated that it is likely that AMPK is active during CHIKV infection because phosphorylation on S108 of the beta subunit of the AMPK complex (PRKAB1), which is known to stimulate AMPK activity [56], was significantly increased at 8 and 12 h p.i. (log2 ratio 0.95 and 0.69). siRNA-mediated AMPK knockdown in combination with nsP2-nCPE overexpression was used to study AMPK’s involvement in the induction of eEF2 phosphorylation. In Vero E6 cells only the siRNA pool targeting AMPKα2 resulted in AMPKα knockdown. While the AMPK pathway can activate eEF2K and possibly also directly phosphorylate eEF2 [51], siRNA-mediated AMPKα2 knockdown could not prevent eEF2 phosphorylation when nsP2-nCPE was overexpressed (Fig 8A).

### siRNA knockdown of PKA reduces (p)eEF2 induction during alphavirus infection

PKA is a heterotetrametric complex consisting of two catalytic subunits and two regulatory subunits. The regulatory subunits have domains to bind cAMP which results in activation of the catalytic subunits. There are 3 isoforms of the catalytic subunit [57]. The substrate sequence motifs that are targeted by PKA are RRxS/T, RKxS/T, KRxS/T or KKxS/T [58]. In the phosphoproteomics experiment (Fig 1), we identified 34 significantly modulated sites with motif RxxS. NetPhorest 2.1 analysis identified six RxxS motifs that had PKA in the top-2 of predicted kinases, with five of those motifs containing RRxS, the most frequently observed target for PKA. For four of these phosphosites an increase in phosphorylation was observed during CHIKV infection, while two showed a decrease (Table 2), suggesting that PKA may become activated during alphavirus infection.

**Table 2:** Peptides with a significantly modified phosphorylation site that is predicted to be a PKA target by NetPhorest 2.1.

Protein	Accession number	Motif sequence	Modified residue (#)	NetPhorest 2.1 prediction (score)	Log2 ratio
ARHGEF2	Q92974	GLFRSES(ph)LESPRG	S617	• Kinase PKA group (0.29) • ACT2/2B TGFbR2 group (0.23)	-1.52 (12 h p.i.)
LRRFIP2	C9JSU1	NSSRRGS(ph)GDTSSL	S328	• Kinase RKC group (0.32) • Kinase PKA group (0.31)	0.36 (12h p.i.)
MATR3	P43243	RHFRRDS(ph)FDDRGP	S188	• Kinase PKA group (0.34) • Kinase ACT2/2B TGFbR2 group (0.22)	0.98 (8 h p.i.) 1.92 (12 h p.i.)
PTRF	Q6NZI2	GDLRRGS(ph)SPDVHA	S365	• Kinase PKA group (0.35) • Kinase CLK group (0.22)	-1.28 (12 h p.i.)
RTN4	Q9NQC3	APKRRGS(ph)SGSVDE	S181	• Kinase CLK group (0.35) • Kinase PKA group (0.34)	0.43 (12 h p.i.)
RPS6	A2A3R5	AKRRRLS(ph)SLRAST	S235	• Kinase CLK group (0.36) • Kinase PKA group (0.35)	1.02 (8 h p.i.)

(ph) indicates the preceding amino acid residue was modified by phosphorylation.

^#^ amino acid residue number according to PhosphoSitePlus [23].

Unfortunately, it proved impossible to employ siRNA-mediated knockdown of these catalytic subunits to study the role of PKA in the induction of eEF2 phosphorylation in response to nsP2-nCPE overexpression, as was done for AMPK above. Compared to control cells transfected with the non-targeting pool (NTP) of siRNAs, nsP2-nCPE expression was severely reduced when one of the catalytic isoforms of PKA was targeted (S1A Fig). Since reduced nsP2 expression translates into less eEF2 phosphorylation, we could not exclude that the reduced level of phosphorylation observed in PKA knockdown cells was solely due to the lower amount of nsP2 in these samples. This unexplained expression issue was not specific for nsP2-nCPE, as it was also observed upon attempts to overexpress other proteins in cells treated with siRNAs targeting PKA-Cα (S1B Fig). However, siRNA-mediated knockdown of PKA followed by SFV infection almost completely prevented the induction of eEF2 phosphorylation (Fig 8B). The amount of viral protein that could be detected in the PKA knockdown sample resulted in clearly observable eEF2 phosphorylation in previous experiments which suggests that knockdown of PKA prevents the induction of eEF2 phosphorylation (Figs 2B and 8B).

### The alphavirus nsP2-NTPase inhibits translation

**Fig 9:**
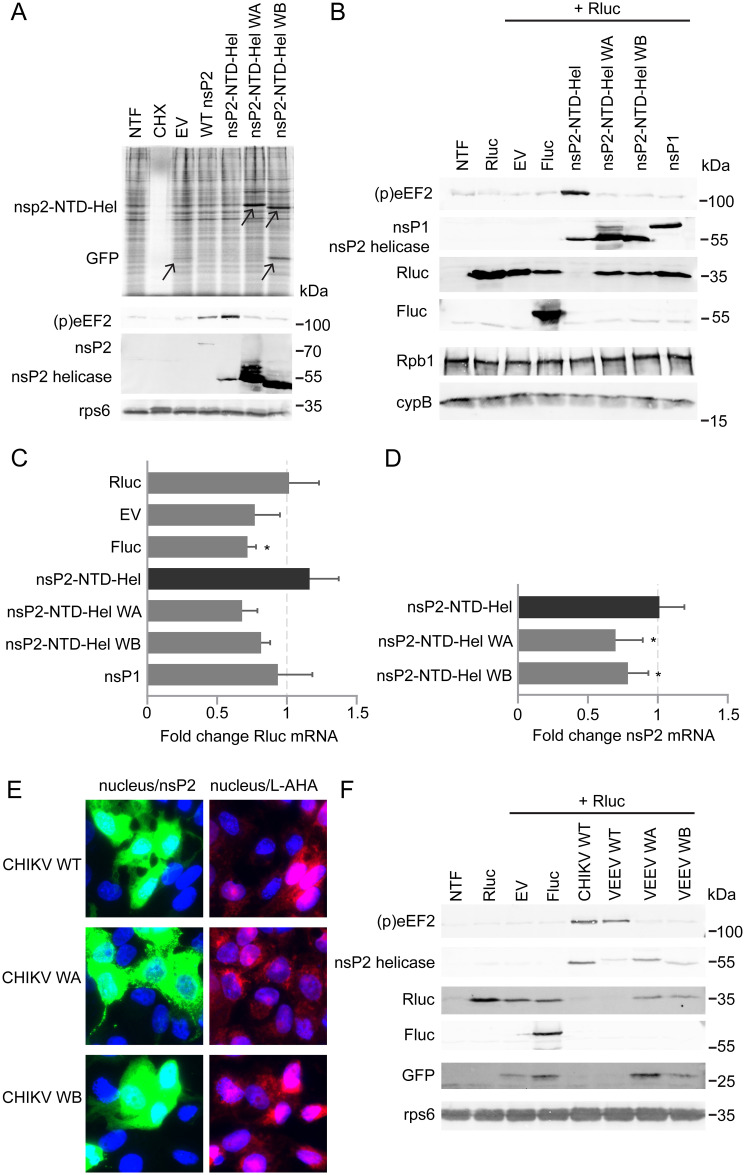
The helicase-associated NTPase domain of nsP2 inhibits translation. (*A*) Top: 293T cells transfected with the pCAGGS-IRES-GFP construct containing the nsP2-NTD-Hel WT sequence or the Walker A and Walker B motif mutants and metabolically labeled with ^35^S-methionine and ^35^S-cysteine for 30 min. Cells treated with 50 μg/ml cyclohemixide (CHX) and cells transfected with an empty vector and non-transfected cells (NTF) were included as controls. Cell lysates were separated by SDS-PAGE and a Storage Phosphor screen was exposed to the dried gel. Bottom: Non-radioactively labeled cell lysates were harvested in parallel and (p)eEF2 (T56) and CHIKV nsP2 helicase were detected by WB. A representative gel is shown of three biological replicates. (*B*) 293T cells were transfected with phRL-TK, expressing *Renilla* luciferase, and co-transfected with the pCAGGS-IRES-GFP construct containing the WT nsP2-NTD-Hel sequence or the Walker motif mutants. Non-transfected cells or cells co-transfected with an empty vector or the pCAGGS-IRES-GFP construct containing the Firefly luciferase or CHIKV nsP1 sequence were used as controls. (p)eEF2 (T56), CHIKV nsP2 helicase, CHIKV nsP1, *Renilla* luciferase (Rluc), Firefly luciferase (Fluc) and RNA polymerase II subunit A (Rpb1) were detected by WB. Representative blots are shown of 3 biological replicates. (*C*) 293T cells were transfected with pRL-TK, expressing Renilla luciferase, and co-transfected with the pCAGGS-IRES-GFP construct containing the WT nsP2-NTD-Hel sequence or the Walker motif mutants. Cells co-transfected with an empty vector or the pCAGGS-IRES-GFP construct containing the Firefly luciferase or CHIKV nsP1 sequence were used as controls. qRT-PCR was used to determine the fold change of Rluc mRNA expression relative to the Rluc only transfection. The nonparametric Mann-Whitney test was used to determine statistical significance (p<0.0.5). The average and standard deviation of 4 biological replicates with each 3 technical replicates is shown. (*D*) 293T cells were transfected with pRL-TK, expressing Renilla luciferase, and co-transfected with the pCAGGS-IRES-GFP construct containing the WT nsP2-NTD-Hel sequence or the Walker motif mutants. Cells co-transfected with an empty vector or the pCAGGS-IRES-GFP construct containing the Firefly luciferase or CHIKV nsP1 sequence were used as controls. qRT-PCR was used to determine the fold change of nsP2 mRNA expression relative to the nsP2-NTD-Hel transfection in the samples in which nsP2 was expressed. The nonparametric Mann-Whitney test (p<0.05) was used to determine statistical significance. The average and standard deviation of 4 biological replicates with each 3 technical replicates is shown. *(E)* Vero E6 cells were transfected with the pCAGGS-IRES-GFP construct containing the WT nsP2-NTD-Hel sequence or the Walker A or Walker B motif mutants and metabolically labeled with L-AHA for 1h. L-AHA incorporation (red) was visualized with a Click azide/alkyne reaction, nsP2 was immunolabeled (green) and nuclear DNA (blue) was stained with Hoechst-33342. Overlays of nucleus/nsP2 and nucleus/L-AHA staining are also shown. Representative images are shown of 2 independent biological replicates. Images of the individual channels from which the overlays were created can be found in S2 Fig. *(F)* 293T cells were transfected with phRL-TK, expressing Renilla luciferase, and co-transfected with the pCAGGS-IRES-GFP construct containing the CHIKV or VEEV WT nsP2-NTD-Hel sequence or the VEEV Walker motif mutants. Non-transfected cells or cells co-transfected with an empty vector or the pCAGGS-IRES-GFP construct containing the Firefly luciferase were used as controls. (p)eEF2 (T56), CHIKV nsP2 helicase, HA-tagged VEEV nsP2, Renilla luciferase (Rluc), Firefly luciferase (Fluc) and GFP were detected by WB. Representative blots are shown of 3 biological replicates.

IRES-driven GFP expression from pCAGGS-IRES-GFP constructs was reduced or even undetectable when using constructs that induce eEF2 phosphorylation, even those lacking nsP2’s C-terminal domain, which is responsible for the known host shut-off mechanisms in Old World alphaviruses [34,36], or those with the nCPE mutations (see above and Fig 6A and 6B). We, therefore, wondered whether eEF2 phosphorylation might constitute an additional mechanism underlying the alphavirus-induced host translational shut-off. To study the effect of CHIKV nsP2 expression on cellular translation in general, nsP2-NTD-Hel and the WA and WB mutants were overexpressed in 293T cells followed by metabolic ^35^S-labeling of newly synthesized proteins. There was no apparent reduction in incorporation of ^35^S-label when nsP2-NTD-Hel was expressed compared to the Walker mutants or the empty vector (Fig 9A). However, we cannot exclude that our analysis was hampered by an insufficient transfection efficiency, as (partial) inhibition could only be observed in transfected cells and may be overshadowed by unchanged protein synthesis in non-transfected cells. However, we did notice, in every experiment, that expression of the WA and WB mutants was much higher than expression of the WT nsP2-NTD-Hel construct, even though the same amount of plasmid DNA was transfected. In the metabolic labeling experiment, additional bands could be observed upon expression of mutant proteins (Fig 9A, arrowheads). We believe these to be nsP2-NTD-Hel and GFP, which were not visible upon WT protein expression. WT protein expression was, however, confirmed by WB using samples that were harvested in parallel (Fig 9A). If this difference in expression efficiency between the WT and mutant constructs is due to the fact that eEF2 phosphorylation only occurs upon WT protein expression, expression of other (host) proteins is likely affected as well.

To specifically analyze transfected cells (expressing nsP2) we determined the effect of CHIKV nsP2-NTD-Hel expression on translation of an unrelated mRNA. CHIKV nsP2-NTD-Hel and the WA and WB mutants were co-transfected with a plasmid encoding *renilla* luciferase (Rluc). Plasmids expressing firefly luciferase (Fluc) or CHIKV nsP1 were included as negative controls, as these proteins were not expected to affect translation of Rluc. Compared to transfection of the Rluc plasmid alone, co-transfection of the empty vector, firefly luciferase, or CHIKV nsP1 construct reduced Rluc expression to a certain extent (likely due to nonspecific competition for cellular resources), but the protein could hardly be detected when nsP2-NTD-Hel was expressed (Fig 9B). Rluc expression could be rescued to the level observed upon Fluc co-expression when cells were co-transfected with the WA and WB mutants, suggesting that eEF2 phosphorylation may indeed contribute to the host translational shut-off during alphavirus infection (Fig 9B). Given that there was no apparent reduction in general protein labeling when nsP2-NTD-Hel was expressed (Fig 9A), we wondered whether there was also an additional shut-off mechanism at the level of transcription in play. This would have a larger effect on a newly introduced construct like Rluc, which still needs to be transcribed, than on a cellular protein for which the mRNA was already present and thus would better explain the level of inhibition observed during nsP2-NTD-Hel expression. An intact helicase domain was previously shown to be required for the nsP2-induced degradation of rpb1, one of the subunits of RNA polymerase II, and this feature is part of the host transcriptional shut-off [34]. However, Akhrymuk et al. showed that mutations in the C-terminal domain of nsP2 were also sufficient to prevent rpb1 degradation, suggesting that both domains are required to induce this part of the host transcriptional shut-off [34,36,37]. In line with these findings, we did not observe degradation of rpb1 when nsP2-NTD-Hel was expressed, suggesting that the reduction in Rluc expression occurs through a different mechanism (Fig 9B). To exclude that the protein expression difference was caused by a difference in mRNA expression, qRT-PCR was used to determine the relative fold change of Rluc and nsP2 mRNA levels, compared to Rluc only and nsP2-NTD-Hel, respectively. For the Fluc sample, the amount of Rluc mRNA appeared to be lower than in the Rluc only sample, but for all other conditions the amount of Rluc mRNA was comparable (Fig 9C). For nsP2, the amount of mRNA was lower in both Walker mutant samples compared to wild type (Fig 9D). These results indicated that the reduced Rluc and nsP2 expression could not be explained by reduced mRNA levels in the nsP2-NTD-Hel sample. It is possible that the translational inhibition through eEF2 phosphorylation mostly affects newly transcribed mRNAs, which would be useful during infection to prevent expression of proteins involved in the innate immune response.

The translational inhibition of proteins expressed from co-transfected plasmids in cells transfected with CHIKV nsP2 was not reflected in the (bulk) analysis of ^35^S-methionine and ^35^S-cysteine incorporation in CHIKV nsP2-transfected cells. We hypothesized that this was because the effect was overshadowed by the metabolic labeling of translation in non-transfected cells. We, therefore, analyzed translation in individual cells by metabolic labeling with L-Azidohomoalanine (L-AHA) and immunofluorescence microscopy. Vero E6 cells grown on coverslips were transfected with the WT or the Walker motif mutant variants of CHIKV nsP2-NTD-Hel. After metabolic labeling of translation with L-AHA, its incorporation was visualized through a Click azide/alkyne reaction using DIBO-Alexa Fluor 555 and fluorescence microscopy. Cells with nsP2-NTD-Hel expression were visualized through immunostaining. The cells that stained positive for CHIKV nsP2 in the WT nsP2-NTD-Hel transfected sample showed less intense staining for L-AHA than surrounding non-transfected cells. This suggests that overall, translation in these cells was inhibited. For the Walker motif mutants there was no difference between L-AHA incorporation between transfected and non-transfected cells (Fig 9E and S2 Fig).

The known host shut-off mechanisms for New World alphaviruses like VEEV are different from those of Old World alphaviruses. VEEV induces a transcriptional shut-off through its capsid protein instead of nsP2 [59,60]. The host translational shut-off induced by VEEV, however, has been linked to nsP2, but it is not yet known which part of nsP2 is responsible [61]. Since we observed induction of eEF2 phosphorylation after VEEV infection (Fig 2B), we wondered whether this could also be regulated through the NTPase in VEEV nsP2-NTD-Hel. To verify this, VEEV nsP2-NTD-Hel WT and Walker A and B mutants with an N-terminal HA-tag for detection in WB were co-expressed with the Rluc plasmid. The WT CHIKV-nsp2-NTD-Hel construct was included as a control. eEF2 became phosphorylated after transfection with the two WT constructs but not with the two VEEV Walker motif mutants. Rluc could hardly be detected after transfection with the WT constructs, but was expressed after transfection with the Walker motif mutants. In the two Walker motif mutant samples, GFP, expressed from the IRES in the same construct, could also be detected, while hardly any GFP was synthesized from the WT constructs. These observations suggest that the host translational shut-off mechanism through an increase in cAMP induced by nsP2 is shared between Old World and New World alphaviruses (Fig 9F).

## Discussion

To gain more insight into signaling pathways that are triggered or inhibited during CHIKV infection, we have performed a phosphoproteomics study and quantified changes in a large number of phosphorylation sites on cellular proteins at 2, 8 and 12 h p.i. Among the host proteins analyzed, eEF2 showed the most prominent change in phosphorylation status following CHIKV infection. The increase could already be detected by 2 h p.i. and is thus an early event during infection. eEF2 phosphorylation leads to its inactivation and inhibition of translation elongation.

The strong induction of eEF2 phosphorylation was also observed during infections with three other alphaviruses, SFV, SINV and VEEV, and the picornavirus CVB3, and was previously reported to occur during Rift Valley Fever virus (RVFV) infection [62], suggesting that the underlying mechanism may be more general. However, phosphorylation was not triggered during infections with two other viruses, EAV and HAdV. The peptide containing eEF2 T56 was not identified in most of the other phosphoproteomics studies on cells infected with human immunodeficiency virus (HIV)-1, Sendai Virus, porcine reproductive and respiratory syndrome virus (PRRSV), influenza A virus, japanese encephalitis virus, west nile virus, vesicular stomatitis virus and blue tongue virus [63–71]. In two HIV-1 studies, the peptide was identified, but the changes were not significant, possibly due to the early time (<1 h) post infection analyzed [72,73].

Other groups have reported a decrease in eEF2 abundance during CHIKV infection [74–76]. However, in our previous proteomics study the abundance of eEF2 did not change [14], nor were we able to reproduce this decrease in eEF2 abundance in our WB analysis in this study. In these other studies, samples were taken much later in infection (24 h p.i. or later) and eEF2 may have been degraded during this stage of infection. This may not be an eEF2-specific property, however, since alphaviruses induce a host shut-off [15]. Two of the studies that reported a decrease in eEF2 levels used 2D-DIGE and most likely the large increase of phosphorylated eEF2 during CHIKV infection significantly decreased the amount of eEF2 in the spot containing the non-phosphorylated form of eEF2, which could explain the observed apparent decrease in abundance in these studies [75,76].

It has been reported that ribosomes and eEF2 co-localize with aggregation sites of the C protein during SINV infection [77]. eEF2 was also identified as an interaction partner of CHIKV nsP3 in a yeast two-hybrid assay [48] and as an interaction partner of SINV nsP3 during infection with a nsP3-GFP expressing SINV mutant [78]. In these studies, the phosphorylation status of eEF2 was not assessed, but it would be interesting to determine whether these interactions occur with the modified or unmodified form of eEF2.

### Regulation of eEF2 phosphorylation during CHIKV infection

Expression of CHIKV or VEEV nsP2-NTD-Hel was sufficient to induce eEF2 phosphorylation. The truncated nsP2-NTD-Hel protein retains NTPase and RTPase activity, but the helicase activity requires the C-terminal domain [49]. Mutation of the Walker A or Walker B motifs in nsP2-NTD-Hel rendered the protein incapable to induce eEF2 phosphorylation, despite the higher expression levels of these mutant proteins compared to WT nsP2-NTD-Hel. Previously, mutagenesis of the WA and WB motifs of the alphavirus helicase was reported to completely abolish the NTPase activity, while there might still be residual RTPase activity [41,49,50]. We, therefore, consider it most likely that the NTPase activity is responsible for triggering eEF2 phosphorylation during alphavirus infection. We also observed eEF2 phosphorylation during infection with the picornavirus CVB3, expressing protein 2C that contains NTPase activity, although 2C belongs to a different helicase superfamily [79,80]. The nsp10-helicase of the arterivirus EAV, on the other hand, does belong to helicase superfamily 1 [81], like the alphavirus nsP2 helicase, but EAV infection did not result in eEF2 phosphorylation. During infection with RVFV, a–ssRNA virus of the bunyavirus family, eEF2 also became phosphorylated [62] but currently there is no evidence for a helicase being encoded in the RVFV genome.

Our study uncovered that expression of an active form of the alphavirus NTPase and the induction of eEF2 phosphorylation are associated with a decrease in ATP and an increase in cAMP concentrations. During cellular stress, an increased AMP:ATP ratio activates the AMPK pathway, resulting in the general inhibition of ATP-consuming (anabolic) processes, including protein synthesis, and the activation of ATP-generating (catabolic) processes [82]. However, in our CHIKV nsP2 overexpression experiments, siRNA-mediated knockdown of AMPK’s catalytic subunits could not prevent eEF2 phosphorylation. Despite the likely increase in AMPK activity during alphavirus infection, our data indicate that eEF2 phosphorylation in infected cells is not mediated via AMPK activation. During RVFV infection, eEF2 phosphorylation still occurred in AMPK-knockout cells and thus also was independent of AMPK activation [46], although at this point we cannot prove that the mechanism of phosphorylation induction by these diverse viruses is triggered by the same stimulus. Based on our findings, we believe it is most likely that during alphavirus infection the observed increase in cAMP levels, a catabolic signal that inhibits protein synthesis, (indirectly) stimulates eEF2K activity. The increase in cAMP occurs much sooner in infection than the decrease in ATP, which matches the 2 h p.i. increase in eEF2 phosphorylation detected in the proteomics experiment. The observed drop in ATP concentration later during infection could then be the result of the increased conversion of ATP into cAMP by a (cellular) adenylyl cyclase (AC) [46] activated by nsP2 or a general increase in ATP consumption due to the infection. An increase in cAMP levels triggers PKA, resulting in eEF2K activation [51]. siRNA knockdown of the PKA catalytic subunits followed by SFV infection resulted in almost undetectable levels of eEF2 phosphorylation making it likely that the activation of eEF2K and subsequent phosphorylation of eEF2 is mediated through PKA.

### A novel host translational shut-off mechanism

Shutting-off host protein synthesis is an important mechanism to redirect resources towards viral replication and to prevent detrimental host responses like activation of the innate immune response [83]. Here, we have shown that the active form of the alphavirus nsP2 NTPase inhibits host translation, independent of the previously identified alphavirus mechanisms to induce transcriptional and translational host shut-off. In this respect, it is noteworthy that eEF2 phosphorylation is also induced by VEEV, since the host transcriptional shut-off during New World alphavirus infection is mediated through C instead of nsP2 [38].

Many viruses block the protein kinase R (PKR) pathway that is activated in response to the sensing of viral dsRNA, to prevent the translational shut-off caused by PKR’s phosphorylation of eukaryotic initiation factor 2α (eIF2α) [84]. During alphavirus infection, however, eIF2α phosphorylation is not prevented and translation of the alphavirus subgenomic mRNA is insensitive to eIF2α phosphorylation [35,83]. The general alphavirus-induced host translational shut-off also occurs independent of PKR and eIF2α phosphorylation, which suggests that host cell translation may also be modulated via other mechanisms [55,83,85]. We propose that the nsP2-induced increase in intracellular cAMP level and the subsequent eEF2 phosphorylation are part of a PKR-independent mechanism of translation inhibition during alphavirus infection. We believe that the translational inhibition mediated through phosphorylation of eEF2 plays a role in this mechanism, but other aspects of protein expression may be affected as well. cAMP is a second messenger and there are several other cAMP sensors besides PKA that may become activated, such as exchange protein directly activated by cAMP (EPAC) [56], cyclic nucleotide-gated (CNG) ion channels [86], or Popeye domain containing (Popdc) proteins [87]. Additionally, PKA has many other downstream targets that could also affect translation [88]. The specificity, duration and intensity of responses to cAMP are modulated via scaffolding proteins that unite ACs, cAMP sensors, activators, effectors and kinase substrates in distinct cellular compartments [88,89]. In this respect, an interesting protein for follow-up studies would be AKAP2, which showed significantly increased phosphorylation on S393 at 12 h post CHIKV infection (Log_2_ ratio 0.34).

Since we observed an overall reduction of protein synthesis upon expression of CHIKV nsP2-NTD-Hel, it will be interesting to find out if viral mRNAs escape the eEF2 phosphorylation-induced shut-off. When eEF2 is depleted from cells, ribosomes become sequestered on mRNA creating a shortage of free ribosomes and preferential translation of mRNAs with high initiation rates [90]. There is already evidence that the alphavirus subgenomic mRNA is translated more efficiently than the genomic mRNA during host shut-off which has been linked to their 5’ UTR sequences [91]. Viral mRNAs are very abundant during infection and if translation is initiated more efficiently than on most cellular mRNAs this may increase their chance of being translated when there is less active non-phosphorylated eEF2 available for translation elongation. It has been described that eIF3, eEF2 and ribosomes concentrate in regions of viral replication [77]. If this concerns the non-phosphorylated form of eEF2 this may also aid efficient translation of viral mRNAs.

Further studies are also needed to elucidate how expression of a viral NTPase results in an increase of the intracellular cAMP concentration. The cAMP concentration can increase either due to increased production by one or more of the 10 adenylyl cyclases or decreased degradation by one or more of the 11 cyclic nucleotide phosphodiesterase (PDE) protein families. Mammalian cells express 9 transmembrane ACs (mACs) isoforms (AC1–9) and a soluble AC (sAC or AC10) [92]. Specific AC isoforms localize to different cellular compartments where they form complexes with distinct activating proteins and downstream effectors. ACs 3, 5, 6 and 8 associate with caveolar and lipid raft microdomains. The other mACs localize to non-raft plasma membrane domains. All of the mACs can be activated by the Gαs protein of G protein-coupled receptors (GPCRs) and GPCRs can be activated by many different ligands. Several ACs can also be activated through Ca2+/Calmodulin, protein kinase C or Raf kinase, and the sAC can be activated by bicarbonate [88,92]. The PDE families and isoforms have different expression patterns, biological functions and specificity for cAMP and cGMP. PDE1, 2, 3 and 10 hydrolyze both cAMP and cGMP, while PDE4, 7 and 8 are highly specific for cAMP [93]. PDE localization is mostly cytosolic and can be targeted to specific compartments within the cell. The PDE activity is regulated through many different pathways and stimuli [93,94]. The next questions to answer are whether ACs are activated or PDEs inhibited by nsP2 and which of these proteins are involved in increasing the cAMP concentration during infection. This should then aid in elucidating how the nsP2 NTPase activity affects the intracellular cAMP concentration and eEF2 phosphorylation.

## Conclusion

**Fig 10:**
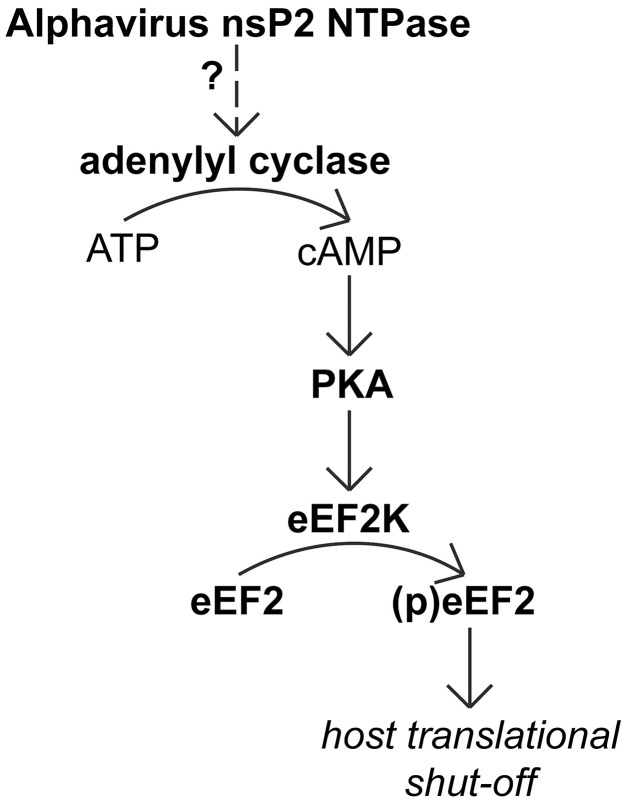
Proposed model for eEF2 phosphorylation during alphavirus infection. The NTPase activity of alphavirus nsP2 induces an increase in the intracellular cAMP concentration which results in activation of PKA and increased eEF2K activity, increased phosphorylation of eEF2 on T56 and T58 and inhibition of translation which contributes to the host shut-off during infection.

Our study revealed that, during the earliest stages of infection, the alphavirus nsP2 NTPase triggers an increase in the intracellular cAMP concentration which results in activation of PKA and increased phosphorylation of eEF2 on T56 and inhibition of translation. We propose that this pathway is responsible for the part of the Old and New World alphavirus-induced translational host shut-off for which a mechanistic explanation was lacking thus far (Fig 10).

## Materials & Methods

### Cells and viruses

MRC-5 cells (human lung fibroblasts, ATCC-CCL-171) were cultured essentially as described previously [30]. Vero E6 cells (African green monkey kidney epithelial cells), 293/ACE2 cells (originally described to be derived from human HEK293 cells [95], but most likely from nonhuman primate origin [14]) and BHK-21 cells (baby hamster kidney fibroblasts) were cultured essentially as described previously [21]. Vero cells (ATCC-CCL81) cells were cultured essentially as described previously [96]. All culture media contained 100 IU/ml penicillin and 100 ug/ml streptomycin unless otherwise specified.

CHIKV LS3 is a synthetic Chikungunya virus based on the consensus sequence of E1-226V isolates [21]. Infections with CHIKV LS3 and LS3-GFP were performed essentially as described previously [21]. The Chikungunya replicon was derived from CHIKV LS3 by replacing the structural genes with a puromycin resistance/foot-and-mouth disease virus 2A oligopeptide/green fluorescent protein (PAC-2A-GFP) reporter gene. The sequence of the 2A oligopeptide contains an amino acid motif that prevents formation of the peptide bond between glycine and the final proline of the sequence which allows expression of multiple proteins from a single ORF [97,98].

Vero E6 cells were infected with the Sindbis virus (SINV) HR-strain [99], Semliki Forest virus (SFV) strain SFV4 [100] or VEEV vaccine strain TC-83 [101] at a multiplicity of infection (MOI) of 5 or 10. Vero E6 cells were infected with a GFP-expressing recombinant human adenovirus type 5 (HAdV-GFP/LUC; [102]) at an MOI of 5 and with a GFP-expressing recombinant coxsackie B3 virus (CVB3) (a kind gift from prof. dr. Frank van Kuppeveld, Utrecht University) at an MOI of 1. BHK-21 cells were infected with the equine arteritis virus (EAV) Bucyrus strain at MOI 5 at 39.5°C essentially as described previously [103].

### Expression plasmids

All oligonucleotide sequences used are listed in S3 Table. All constructs were verified by sequence analysis. IRES-eGFP was amplified from pL-eGFP, a plasmid with a T7 promoter in which the foreign gene is under control of an encephalomyocarditis virus internal ribosomal entry site and inserted in plasmid pCAGGS/MCSII [104] using *XhoI* and *BglII* restriction sites using standard cloning techniques to create pCAGGS-IRES-GFP. The nucleotide sequences of CHIKV nsPs and protein domains were amplified from pCHIKV-LS3 [21] and pCHIKrepLS3-nCPE, a replicon derivative from pCHIKV-LS3 mutated in nsP2 (P718S, K649D and R650H), and inserted into pCAGGS-IRES-GFP using *EcoRI* or *SacI* and *XhoI*. Firefly luciferase was amplified from pGL3-MKP-1-Luc [105] and inserted into pCAGGS-IRES-GFP using *EcoRI* and *XhoI*.

pCAGGS-VEEV-nsP2-NTD-Hel was created by amplifying the nsP2-NTD-Hel sequence from cDNA prepared from the VEEV TC-83 stock. The PCR oligonucleotides introduced a start codon, N-terminal HA-tag, a stop codon and SacI and XhoI restriction sites to use for cloning.

pCAGGS-CHIKV-nsP2-NTD-Hel and pCAGGS-VEEV-nsP2-NTD-Hel Walker A and B mutants were created by cloning the CHIKV-nsP2-NTD-Hel or VEEV-nsP2-NTD-Hel PCR product into pCR2.1-TOPO (Thermo Fisher Scientific) for site-directed mutagenesis to introduce the Walker A and B-inactivating mutations prior to transferring the mutated sequences to pCAGGS-IRES-GFP. *Renilla* luciferase was expressed from phRL-TK or pRL-TK (both Promega).

pCAGGS-FLAG-eEF2 was created by cloning the human synthetic FLAG-eEF2 (IDT) sequence into pCAGGS-MCSII [104]. pCMV-FLAG-Ub expressed FLAG-tagged ubiquitin [106]. pCDNA3-FLAG-UbcH10 was a kind gift from prof. Akira Nakagawara (Chiba Cancer Centre, Japan) [107].

### SILAC labeling and CHIKV infection of SILAC-labeled cells

MRC-5 cells were cultured in SILAC DMEM (PAA) containing 10% dialyzed Fetal Bovine Serum (FBS) (Gibco), 0.280 mM arginine, 0.384 mM lysine, 0.5 mM proline, 10 mM HEPES, 2 mM L-Glutamine and 1% NEAA for >5 cell doublings to ensure complete incorporation of labeled amino acids. Arginine to proline conversion was not observed under these conditions. The ‘light SILAC labeling’ was performed using Arg ^12^C_6_^14^N_4_ and Lys ^12^C_6_^14^N_2_, whereas the ‘heavy SILAC sample’ was labeled with Arg ^13^C_6_^15^N_4_ and Lys ^13^C_6_^15^N_2_ (Cambridge Isotope Laboratories).

SILAC-labeled MRC-5 cells were seeded in 75-cm^2^ flasks 1 day before infection with CHIKV-LS3 [21] at MOI 5. One hour post infection (h p.i.), the inoculum was removed and replaced with SILAC DMEM containing 2% dialyzed FBS, 0.280 mM arginine, 0.384 mM lysine, 0.5 mM proline, 25 mM HEPES, 2 mM L-Glutamine and 1% NEAA. At 2, 8, and 12 h p.i., infected and mock-infected cells were harvested for phosphoproteomics analysis by lysis in 4% SDS, 0.1M Tris pH 7.6, followed by heating to 96°C for 10 min. At 12 h p.i,. protein lysates for western blot (WB) analysis were harvested in 4× Laemmli sample buffer (LSB) (100 mM Tris-HCl, pH 6.8, 40% glycerol, 8% SDS, 40 mM DTT, 0.04 mg/ml bromophenol blue) and cells grown on coverslips were fixed in 3% PFA in PBS. The experiment was performed in duplicate with a label swap (Fig 1A).

### Protein digestion

The protein concentration of the SILAC cell lysates was determined using the bicin chonicic acid assay (Pierce). Digestion of the proteins was performed using the Filter-Aided Sample Preparation (FASP) method [108], for which equal amounts (900 μg) of mock- and virus-infected cell lysates were mixed and DTT was added to a final concentration of 50 mM, followed by a 5-min incubation at 70°C. Samples were loaded on two 15-ml 30 kDa Microcon filter devices (Millipore), which were washed twice with 8 M urea 0.1 M Tris pH 8.5, while cysteines were alkylated with 50 mM iodoacetamide in the same buffer. Samples were washed 3 times with 8 M urea, 0.1 M Tris pH 8. Proteins were digested overnight at room temperature using 20 ug endoLysC (Wako Pure Chemical Industries) in the same buffer per filter device. The sample was diluted fourfold with 50 mM ammonium bicarbonate pH 8.4 containing 20 ug trypsin (Worthington Chemical Corporation), and digested for 4 h at room temperature. Peptides were collected by centrifugation, acidified to a final percentage of 1% TFA, and desalted using solid phase extraction. Peptides were eluted in 20/80/0.1 (v/v/v) of milliQ/acetonitrile (ACN) (Actu-All Chemicals)/trifluoric acid (TFA) (Sigma-Aldrich).

### Phosphopeptide enrichment

The samples were enriched for phosphopeptides by Hydroxy Acid Modified Metal Oxide Chromatography (HAMMOC) [109]. Per 500 ug peptide digest a 200 ul tip with an Octyl C8 membrane (Empore) and 2.5 mg Titansphere TiO_2_ 10 μm (GL Sciences) were used. The tips were preconditioned with 20/80/0.1 (v/v/v) milliQ/ACN/TFA (solution A) and equilibrated with 300 mg/ml DL lactic acid (Fluka Analytical) in solution A. Peptide samples were mixed 1:1 with 300 mg/ml DL lactic acid in solution A and loaded on the tips. The tips were washed with 300 mg/ml DL lactic acid in solution A and solution A. 100 ul 20% phosphoric acid (Sigma-Aldrich) was put in collection tubes and phosphopeptides were eluted with 50 ul 0.5% piperidine (Actu-All Chemicals) followed by 50 ul 5% piperidine. Peptides were desalted on 200 ul tips with an SDB-XC membrane (Empore). Tips were preconditioned with solution A and equilibrated with 0.1% TFA. Samples were loaded on the tips and the tips were washed with 0.1% TFA. Peptides were eluted with solution A and lyophilized in a CHRIST RVC-2-18 CDplus.

### Mass spectrometry

Phosphopeptide-enriched samples were analyzed via on-line C18-nano-HPLC-MS with a system consisting of an Easy nLC 1000 gradient HPLC system (Thermo, Bremen, Germany), and a Q-Exactive mass spectrometer (Thermo). Fractions were injected onto a homemade precolumn (100 μm × 15 mm; Reprosil-Pur C18-AQ 3 μm, Dr. Maisch, Ammerbuch, Germany) and eluted via a homemade analytical nano-HPLC column (15 cm × 50 μm; Reprosil-Pur C18-AQ 3 um). The gradient was run from 0% to 30% solvent B (10/90/0.1 (v/v/v) water/ACN/FA) in 120 min. The nano-HPLC column was drawn to a tip of ∼5 μm and acted as the electrospray needle of the MS source. The Q-Exactive mass spectrometer was operated in top10-mode. Parameters were resolution 70,500 at an AGC target value of 3,000,000, maximum fill time of 250 ms (full scan), and resolution 17,500 at an AGC target value of 200,000/maximum fill time of 80 ms for MS/MS at an intensity threshold of 2,500. Apex trigger was set to 1 to 15 seconds, and allowed charges were 2–6. Each sample was analyzed in duplo.

### Mass spectrometry data analysis

Raw data files were analyzed using Maxquant 1.4.0.3 [110] using the Andromeda search engine [111]. Databases used for the main search were UNIPROT/KB_Human (88,665 entries) and a custom-made database containing the protein sequences of CHIKV-LS3 (11 entries) using the GenBank sequence (accession KC149888) [21]. For the first search a smaller database, human.first.search (15,612 entries) containing a subset of human protein sequences was used. A list of common contaminants was included in the search. To reach a false discovery rate (FDR) of 0.01 a concatenated reversed database (KR special amino acids) was used, FDR at the peptide level was also 0.01. Enzyme specificity was Trypsin/P. Variable modifications included in the search were oxidation (M), acetylation (protein N-term) and phospho (STY), whereas carbamidomethyl (C) was included as a fixed modification. Up to three missed cleavages and a maximum of five modifications per peptide were allowed. The minimum score for modified peptides was set to 40 and the minimum delta score for modified peptides was set to 17. Match between runs was turned on with a matching time window of 1 minute. MaxQuant results were further analyzed with Perseus version 1.2.0.17 and Microsoft Excel 2010 and GraphPad Prism version 5.

From the list of identified phosphorylation sites, contaminants and peptides identified with the concatenated reversed database were removed before further analysis. The column “Class I phosphosite” was added to indicate which phosphosites have a localization probability of >0.75 and a probability localization score difference ≥5 [19]. Please note that phosphosite identifications with lower scores may be less reliable. Normalized ratios from one of the biological replicates were inverted to ensure all ratios were displayed as infected/mock. Normalized ratios were log_2_ transformed and averages and variances were calculated for each experiment. Phosphorylation sites with a variance <0.25 were included in the analysis. The list of excluded sites was manually inspected and some of these were still included in the analysis when a large change was observed in both replicates in the same direction but with relatively large variation. Significance B with a Benjamini-Hochberg FDR of 0.05 (both-sided) was calculated in Perseus 1.2.0.17 separately for each time point to determine which phosphorylation sites were significantly modulated during CHIKV infection.

Maximal Motif Finder for Phosphoproteomics datasets was used to determine enrichment of certain motifs in the phosphorylated host peptides that were significantly modulated [22]. A complete analysis was performed with a width of 13 for reconstruction and finding motif, the SwissProt human proteome as background for statistical significance and reconstruction, the range of minimal occurrences of the number of peptides containing the motif from 5–15% and a significance level of 10^−6^.

NetPhorest 2.1 was used to analyze enriched motifs with the RRxS motif to identify potential kinases [112].

### Western blot analysis

Western blot (WB) analysis was performed essentially as described previously [14]. Primary antibodies used were rabbit antisera against CHIKV nsP1, CHIKV nsP2 helicase, CHIKV nsP2 protease, CHIKV nsP3 and CHIKV nsP4 [21], CHIKV capsid protein, SFV capsid protein (all kind gifts from prof. Andres Merits, University of Tartu, Estonia), rabbit polyclonal against eEF2 #2332, mouse monoclonal against STAT-1 (9H2) #9176, mouse monoclonal against Rpb1 CTD (48H) #2629 (all three Cell Signaling Technology), mouse monoclonal against β-actin #A5316; mouse monoclonal FLAG M2 #F1804 (both Sigma), goat polyclonal against cyclophilin B (C15) #sc-20361, mouse monoclonal against ISG15 (F-9) # sc-166755 (both Santa Cruz), rabbit polyclonal against Renilla Luciferase #GTX125851 (GeneTex) and an in-house raised rabbit polyclonal serum a-GFP. Antisera were diluted in 1% casein in phosphate buffered saline containing 0.1% Tween-20 (PBST). Rabbit polyclonal phospho-eEF2 (Thr56) #2331, rabbit monoclonal rps6 #5G10, rabbit polyclonal PKA Cα #4782 and rabbit polyclonal AMPKα #2532 (all Cell Signaling Technology) were diluted in 1% BSA in Tris-buffered saline containing 0.1% Tween-20 (TBST). β-actin, cyclophilin B (cypB) or rps6 were used as a loading control. Biotin-conjugated swine-a-rabbit (DAKO) or goat-a-mouse (DAKO) or goat-a-mouse (Invitrogen) or donkey-a-rabbit (Invitrogen), and Cy3-conjugated mouse-a-biotin #200-162-211 (Jackson Immuno Research) were used for fluorescent detection with a Typhoon-9410 scanner (GE Healthcare) or an Alliance Q9 advanced imager (Uvitec).

### Immunofluorescence microscopy

Immunofluorescence microscopy (IFA) was performed essentially as described previously [14]. Primary antibodies used were rabbit polyclonal against CHIKV E2 [113] and mouse monoclonal antibody J2 against dsRNA (English & Scientific Consulting) diluted in 0.5% BSA in PBS. Primary antibodies were detected with donkey-a-rabbit-Cy3 or goat-a-mouse-Alexa488 (Jackson). Nuclei were stained with Hoechst33342. Coverslips were mounted with Prolong (Invitrogen) and examined using a Zeiss Axioskop2 fluorescence microscope with Axiocam HRc camera and AxioVision software.

### Replicon RNA transfection

BHK-21 cells were transfected by electroporation using 4x10^6^ cells in 400 ul PBS and 4 ug of *in vitro* transcribed capped or uncapped CHIKV replicon RNA per cuvette. After 2 pulses with an Eurogentec Easyjet Plus instrument set at 850 V and 25 μF, cells were taken up in pre-warmed medium, seeded at a density of 6 x 10^5^ cells/10cm^2^ dish, incubated at 37°C, and harvested 6, 8, and 10 h p.i. for WB and in-gel hybridization analysis.

### RNA isolation, denaturing agarose electrophoresis and in-gel hybridization

Total RNA was isolated from cells lysed in 20 mM Tris-HCl (pH 7.4), 100 mM LiCl, 2 mM EDTA, 5 mM DTT, 5% (w/v) lithium dodecyl sulfate, and 100 mg/ml proteinase K as described previously [21]. RNA was separated in 1.5% denaturing formaldehyde-agarose gels using the MOPS buffer system as described previously [114]. RNA molecules were detected by direct hybridization of the dried gel with ^32^P-labeled oligonucleotides essentially as described previously [115]. CHIKV positive- or negative-stranded RNAs were visualized as described previously [21] using probe CHIKV-hyb4 or CHIKV-hyb2, which are complementary to the 3’ end of the genome (detects genome and subgenomic mRNA) and anti-genome (detects negative-stranded RNA), respectively. 18S ribosomal RNA was used as loading control. Storage Phosphor screens were exposed to hybridized gels and scanned with a Typhoon-9410 scanner (GE Healthcare).

### Plasmid DNA transfection

2.5 x 10^5^ 293T cells were seeded in 12-well culture plates and after 24 h transfected with 2 ug plasmid DNA and 5 ul Lipofectamine2000/ml in DMEM containing 10% FCS and 2 mM L-Glutamine. For the Rluc co-transfection experiment 1 ug phRL-TK/ml was transfected. At 16 h p.t. cells were washed with ice cold PBS, lysed in 4× LSB for WB analysis. 1.0 x 10^4^ 293T cells were seeded in 96-well white μclear bottom plates and after 24 h transfected with 2 ug plasmid DNA and 5 ul Lipofectamine2000/ml in DMEM containing 10% FCS and 2 mM L-Glutamine. At 16 h p.t. the cellular ATP and cAMP levels were determined.

Vero E6 cells were transfected with 1 ug plasmid DNA and 3 ul Lipofectamine2000/ml in DMEM containing 8% FCS without antibiotics in 12-well culture plates. At 18 h p.t. cells were washed with ice cold PBS and lysed in 4× LSB for WB analysis.

### Compound treatment

1 x 10^4^ 293T cells were seeded in white μclear 96-well culture plates and 1.4*10^5^ cells were seeded in 12-well culture plates. After 24 h cells were incubated with increasing concentrations of ATP-depleting compounds Antimycin A (Sigma) and 2-deoxy-D-glucose (Sigma) for 30 min at 37°C. ATP levels were determined with the CellTiter-Glo 2.0 assay (Promega) as per the manufacturer’s instructions. Luminescence was measured in a Mithras LB 940 Multilabel reader (Berthold Technologies). Cells were lysed in 4× LSB for WB analysis. An SFV MOI 10 sample 6 h p.i. was included as a positive control.

1 x 10^4^ Vero E6 cells were seeded in white μclear 96-well culture plates. After 24 h the cells were incubated with a 2-fold dilution of the cAMP increasing compound forskolin (Cayman chemical) for 30 min at 37°C and cAMP levels were determined with the cAMP-Glo assay (Promega) as per the manufacturer’s instructions. Luminescence was measured in a Mithras LB 940 Multilabel reader (Berthold Technologies). 1.4*10^5^ cells were seeded in 12-well culture plates. After 24 h cells were incubated with a 2-fold dilution of cAMP increasing compounds forskolin. For the cAMP-Glo Assay forskolin had to be diluted in an induction buffer containing two phosphodiesterase inhibitors to prevent cAMP hydrolysis; 500 μM isobutyl-1-methylxanthine (IBMX) (Sigma) and 100 μM Ro 20–1724 (Sigma). Graphpad Prism 8 was used to do a two-tailed non-paired t-tests to determine significant differences. For the WB experiment the 2-fold dilution was started with 5 μM forskolin, 500 μM IBMX and 100 μM Ro 20–1724. Cells were lysed in 4× LSB for WB analysis. An SFV MOI 10 sample 6 h p.i. was included as a positive control.

### siRNA knockdown

siGenome Human siRNA SMARTpools were ordered from Dharmacon. AMPK: M-005027-02 and M-005361-02. PKA: M-004649-01, M-004650-00 and M-004651-02. 0.2 x 10^5^ Vero E6 cells were seeded in 12-well culture plates and after 24 h transfected with 25 nM siRNA pools with 2 μl Dharmafect1/ml (Dharmacon) in 1 ml DMEM containing 8% FCS without antibiotics. 24 h p.t. the medium was replaced with DMEM containing 8% FCS without antibiotics. 48 h p.t. the cells were used for plasmid DNA transfection or infected with SFV MOI 1.

### Treatment of cells with 5’pppRNA and poly I:C

The *in vitro* synthesis of 5’pppRNA representing sequences from the 5’- and 3’-untranslated regions of the vesicular stomatitis virus (VSV) genome was described previously [30]. MRC-5 cells were transfected with 0.1, 1, or 10 ng/ml 5’pppRNA or control RNA (same sequence but lacking the 5’ppp moiety; Integrated DNA Technologies Inc., IA, USA) 1 h prior to infection or mock infection with CHIKV-LS3-GFP at MOI 0.1, as described previously [30]. At 24 h p.i., cells were lysed in 4× LSB for WB analysis.

1.6*10^5^ MRC-5 cells were seeded in 12-well culture plates and after 24 h transfected with 50, 500 and 5000 ng/ml poly I:C (Sigma) using 2.4 ul Lipofectamine2000/ml or treated with 200U/ml IFNβ (PBL). Cells were incubated at 37°C. At 24 h p.t. cells were lysed in 4× LSB for WB analysis.

### Metabolic labeling

293T cells were transfected with plasmids in 12-well culture plates as described above, 15 h p.t. cells were starved in DMEM lacking L-methionine and L-cysteine (Gibco) for 30 min, and subsequently incubated with 44 μCi EasyTag EXPRESS ^35^S protein labeling mix (PerkinElmer) for 30 min. Total protein samples were analyzed by SDS PAGE as described above. 35S-labeled proteins were detected by drying the gels and exposing them to a Storage Phosphor screen, which was scanned 3 days later with a Storm 820 scanner (GE Healthcare).

Vero E6 cells in 12-well culture plates with coverslips were transfected with 0.5 ug plasmid DNA and 3 ul Lipofectamine2000/ml in DMEM containing 8% FCS without antibiotics. At 18 h p.t. cells were starved in DMEM lacking L-methionine and L-cystine (Gibco) to which 250 μM L-cystine was added for 30 min, and subsequently incubated with 25 μM Click-IT AHA (L-Azidohomoalanine) (Invitrogen) for 1h. Cells were washed twice with warm PBS and fixed for 15 min in 3% PFA in PBS. Cells were permeabilized in 0.2% Triton X-100 in PBS for 15 min and washed with 3% FCS in PBS. Cells were incubated with 15 uM Click-iT DIBO-Alexa Fluor 555 (Invitrogen) in 1% FCS in PBS for 1h at RT in the dark. Cells were washed twice with 1% FCS in PBS and twice with PBS-Glycine for a total of >15 min. Cells were incubated with a rabbit antiserum against CHIKV nsP2 helicase (kind gift from prof. Andres Merits, University of Tartu, Estonia) diluted 1:1000 in 5% FCS in PBS for 1h at 37°C in the dark. Cells were washed 3 times with PBS-Glycine for 10 min. The secondary antibody goat-a-rabbit-Alexa488 (Invitrogen) was diluted 1:300 in 5% FCS in PBS and cells were incubated for 1h at 37°C in the dark. Cells were washed 3 times in PBS for 10 min. Coverslips were mounted with Prolong Glass and analyzed using a Leica DM6B Fluorescence Microscope and LASX software (Leica).

### qRT-PCR

Intracellular RNA was isolated from 293T cells transfected with plasmid DNA using TriPure isolation reagent (Life Technologies) according to the manufacturer’s instructions. The RNA was used to determine relative CHIKV nsP2 and Rluc mRNA levels using internally controlled multiplex quantitative TaqMan real-time PCR. PGK1 mRNA expression levels were monitored to correct for variations in isolation or qRT-PCR efficiency. A 10-μl reaction mixture was composed of 1.25 μl of template RNA, 2.5 μl of TaqMan Fast Virus one-step master mix (Thermo Fisher Scientific), 0.5 μl nsP2 assay (CHIKV-nsP2-Fw, CHIKV-nsP2-Rev & CHIKV-nsP2-probe), 0.5 μl Rluc assay (Rluc-Fw, Rluc-Rev & Rluc-Probe), 0.5 μl PGK1 assay (Thermo Fischer #4448491), and 4.75 μl of nuclease-free water (Sigma). All reactions were performed in triplicate in a 384-well plate using the CFX384 Touch real-time PCR detection system and the following program: 5 min at 50°C and 20 s at 95°C, followed by 46 cycles of 5 s at 95°C and 30 s at 60°C. Data were analyzed with CFX manager 3.1 software (Bio-Rad). For absolute quantification, standard curves were generated using 10-fold serial dilutions of known quantities of in vitro-transcribed CHIKV and Rluc mRNA (from a synthetic minigene).

*The mass spectrometry proteomics data have been deposited to the ProteomeXchange Consortium* (http://proteomecentral.proteomexchange.org) *via the PRIDE partner repository* [116] *with the dataset identifier PXD009381*.

## Supporting information

S1 Fig siRNA knockdown of PKA inhibits plasmid expression. (*A*) Vero E6 cells were transfected with siRNAs targeting the catalytic subunit isoforms of PKA. A non-targeting pool (NTP) of siRNAs was used as a control. 2 d p.t. the cells were transfected with pCAGGS-IRES-nsP2-nCPE and harvested 18 h p.t. Cells transfected with an empty vector and non-transfected cells (NTF) were included as controls. Protein lysates were separated by SDS-PAGE and nsP2, (p)eEF2 (T56), and PKA C-α were detected by WB. (*B*) Vero E6 cells were transfected with siRNAs targeting PKA C-α. A non-targeting pool (NTP) of siRNAs was used as a control. 2 d p.t. the cells were transfected with pCAGGS-IRES-GFP-nsP2-nCPE, pCAGGS-IRES-GFP-nsP1, pCAGGS-FLAG-eEF2, pCMV-FLAG-Ub or pCDNA3-FLAG-UbcH10 and harvested 18 h p.t. Protein lysates were separated by SDS-PAGE and nsP2, nsP1, and FLAG-tag were detected by WB. Plasmid expression differences between NTP and PKA C-α siRNA transfected cells were quantified using ImageQuantTL (GE Healthcare).
(PDF)

S2 Fig nsP2-NTD-Hel expression induces a general translational shut-off. Vero E6 cells were transfected with the pCAGGS-IRES-GFP construct containing the WT or the Walker A or Walker B motif mutant sequences of nsP2-NTD-Hel and metabolically labeled with L-AHA for 1h. L-AHA incorporation (red) was visualized with a Click azide/alkyne reaction, nsP2 was immunolabeled (green) and nuclear DNA (blue) was stained with Hoechst-33342. Non-transfected (NTF) cells treated with CHX were used as a negative control. The individual channels are shown and the insets with magnified overlays of nucleus/nsP2 and nucleus/L-AHA from Fig 9E. The white squares indicate which part of the larger images was used to create the overlays. This experiment was performed twice, representative images are shown.
(PDF)

S1 Table Complete list of identified phosphorylation sites, the first sheet contains all sites identified in host proteins, the second sheet contains the sites identified in CHIKV proteins. (XLSX)

S2 Table Lists with phosphorylation sites that were significantly modulated during CHIKV infection. Each time point is placed in a separate sheet.
(XLSX)

S3 Table List of oligonucleotide sequences. (PDF)
