# Supplementary figures and images for: The alphavirus nonstructural protein 2 NTPase induces a host translational shut-off through phosphorylation of eEF2 via cAMP-PKA-eEF2K signaling
Source: PLoS Pathog. 2023 Feb 27;19(2):e1011179. doi: 10.1371/journal.ppat.1011179 (PMC9997916; doi:10.1371/journal.ppat.1011179)

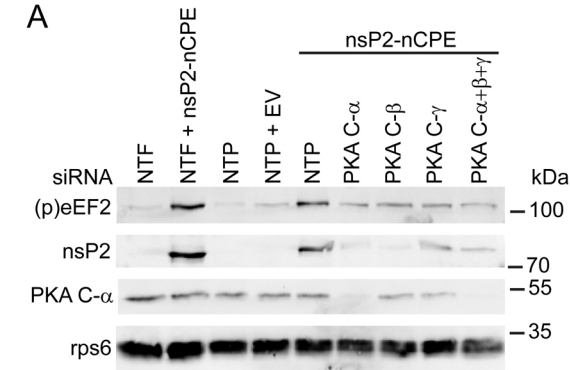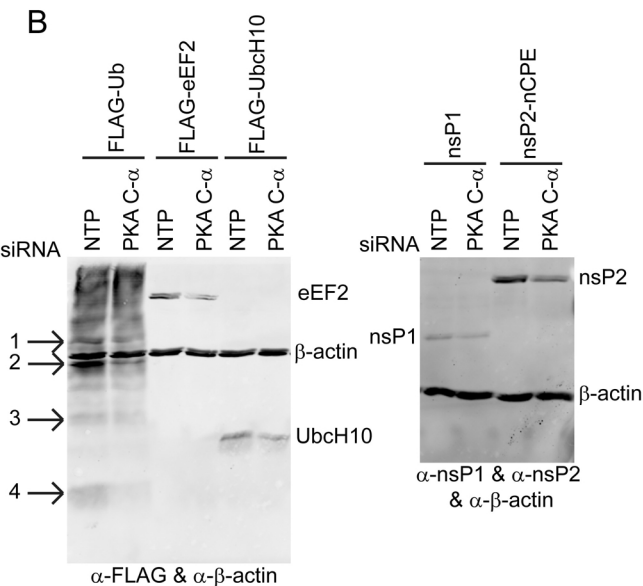

| Protein band | % PKA C- $\alpha$ /NTP |
|--------------|------------------------|
| nsP2         | 55                     |
| nsP1         | 35                     |
| eEF2         | 31                     |
| UbchH10      | 44                     |
| Ub protein 1 | 53                     |
| Ub protein 2 | 32                     |
| Ub protein 3 | 46                     |
| Ub protein 4 | 7                      |

Supplement: S1 Fig — (A) Vero E6 cells were transfected with siRNAs targeting the catalytic subunit isoforms of PKA. A non-targeting pool (NTP) of siRNAs was used as a control. 2 d p.t. the cells were transfected with pCAGGS-IRES-nsP2-nCPE and harvested 18 h p.t. Cells transfected with an empty vector and non-transfected cells (NTF) were included as controls. Protein lysates were separated by SDS-PAGE and nsP2, (p)eEF2 (T56), and PKA C-α were detected by WB. (B) Vero E6 cells were transfected with siRNAs targeting PKA C-α. A non-targeting pool (NTP) of siRNAs was used as a control. 2 d p.t. the cells were transfected with pCAGGS-IRES-GFP-nsP2-nCPE, pCAGGS-IRES-GFP-nsP1, pCAGGS-FLAG-eEF2, pCMV-FLAG-Ub or pCDNA3-FLAG-UbcH10 and harvested 18 h p.t. Protein lysates were separated by SDS-PAGE and nsP2, nsP1, and FLAG-tag were detected by WB. Plasmid expression differences between NTP and PKA C-α siRNA transfected cells were quantified using ImageQuantTL (GE Healthcare). (PDF) [file ppat.1011179.s001.pdf]

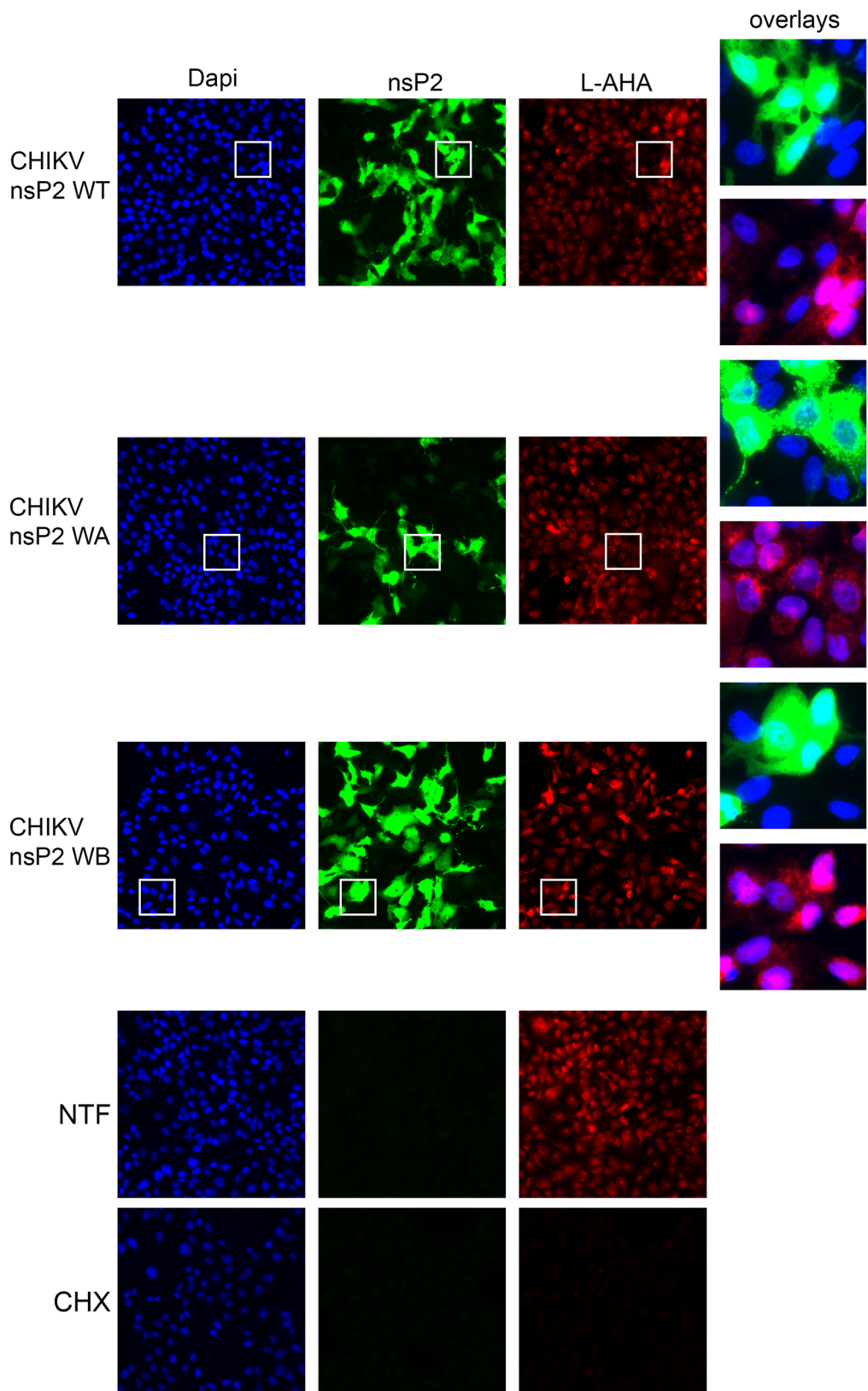

Supplement: S2 Fig — Vero E6 cells were transfected with the pCAGGS-IRES-GFP construct containing the WT or the Walker A or Walker B motif mutant sequences of nsP2-NTD-Hel and metabolically labeled with L-AHA for 1h. L-AHA incorporation (red) was visualized with a Click azide/alkyne reaction, nsP2 was immunolabeled (green) and nuclear DNA (blue) was stained with Hoechst-33342. Non-transfected (NTF) cells treated with CHX were used as a negative control. The individual channels are shown and the insets with magnified overlays of nucleus/nsP2 and nucleus/L-AHA from Fig 9E. The white squares indicate which part of the larger images was used to create the overlays. This experiment was performed twice, representative images are shown. (PDF) [file ppat.1011179.s002.pdf]
