# Supplementary material for: The alphavirus nonstructural protein 2 NTPase induces a host translational shut-off through phosphorylation of eEF2 via cAMP-PKA-eEF2K signaling
Source: PLoS Pathog. 2023 Feb 27;19(2):e1011179. doi: 10.1371/journal.ppat.1011179 (PMC9997916; doi:10.1371/journal.ppat.1011179)
Supplement: S3 Table — (PDF) [file ppat.1011179.s005.pdf]

Oligo	Sequence 5’-3’	5’-tail	Application
IRES-GFP-Fw	ATGGCGACGCTCGAGGCCCCTCTCCCTCCCCCCC	XhoI	Amplification IRES-GFP
IRES-GFP-Rev	ATGGCGACGAGATCTTTACTTGTACAGCTCGTCCATGCCGTG	BglII	Amplification IRES-GFP
CHIKV-nsP1-Fw	GGGCCCTTTGAATTCATGGATCCTGTGTACGTGGACATAGAC	EcoRI	Amplification CHIKV nsP1
CHIKV-nsP1-Rev	GGGCCCTTTCTCGAGTTATGCGCCCGCTCTGTCCTC	stop codon + XhoI	Amplification CHIKV nsP1
CHIKV-nsP2-Fw	GGGCCCTTTGAGCTCATGGGAATAATAGAGACTCCGAGAGGAGC	SacI + start codon	Amplification CHIKV nsP2, CHIKV nsP2-nCPE, CHIKV nsP2-NTD, CHIKV-nsP2-NTD-Hel
CHIKV-nsP2-Rev	GGGCCCTTTCTCGAGTTAACATCCTGCTCGGGTGACCT	stop codon + XhoI	Amplification CHIKV nsP2, CHIKV nsP2-nCPE, CHIKV-nsP2-Hel-Pro, CHIKV nsP2-Hel-Pro-nCPE, CHIKV nsP2-Pro, CHIKV nsP2-Pro-nCPE
CHIKV-nsP3-Fw	GGGCCCTTTGAATTCATGGCACCGTCGTACCGGGTAAAAC	EcoRI + start codon	Amplification CHIKV nsP3
CHIKV-nsP3-Rev	GGGCCCTTTCTCGAGTTACCCACCTGCCCTGTCTAGTCA	stop codon + XhoI	Amplification CHIKV nsP3
CHIKV-nsP4-Fw	GGGCCCTTTGAATTCATGTATATATTCTCGTCGGACACCGGTCC	EcoRI + start codon	Amplification CHIKV nsP3
CHIKV-nsP4-Rev	ATGGCGACGAGATCTTTACTTGTACAGCTCGTCCATGCCGTG	stop codon + XhoI	Amplification CHIKV nsP4
CHIKV-nsP2-NTD-Rev	GGGCCCTTTCTCGAGTTAGCCCACCAGTACCAGTCCTG	stop codon + XhoI	Amplification CHIKV nsP2-NTD
CHIKV-nsP2-Hel-Fw	GGGCCCTTTGAGCTCATGGACTTGACTAATCCGCCCTACC	SacI + start codon	Amplification CHIKV nsP2-Hel, CHIKV nsP2-Hel-Pro, CHIKV nsP2-Hel-Pro-nCPE
CHIKV-nsP2-Hel-Rev	GGGCCCTTTCTCGAGTTATGTATCGAAGGTCATTTGGTGACTG	stop codon + XhoI	Amplification CHIKV nsP2-N-Hel, CHIKV nsP2-Hel
CHIKV-nsP2-Pro-Fw	GGGCCCTTTGAGCTCATGTTCCAAAATAAAGCCAACGTTTG	SacI + start codon	Amplification CHIKV nsP2-Pro, CHIKV nsP2-Pro-nCPE
LUC-Fw	GGGCCCTTTGAATTCATGGAAGACGCCAAAAACATAAAGAAAG	EcoRI + start codon	Amplification firefly luciferase
LUC-Rev	GGGCCCTTTCTCGAGTTACACGGCGATCTTTCCGC	XhoI	Amplification firefly luciferase
CHIKV-nsP2-QC-WalkerA-Fw	ACCGGGATCTGGCGCGTCAGCTATTATCAAG		Site directed mutagenesis CHIKV nsP2-NTD-Hel-WA
CHIKV-nsP2-QC-WalkerA-Rev	CTTGATAATAGCTGACGCGCCAGATCCCGGTACT		Site directed mutagenesis CHIKV nsP2-NTD-Hel-WA
CHIKV-nsP2-QC-WalkerB-Fw	GTGTTGTACGTAGCCGCGGCGTTTGCGTG		Site directed mutagenesis CHIKV nsP2-NTD-Hel-WB
CHIKV-nsP2-QC-WalkerB-Rev	AGTGGCACGCAAACGCCGCGGCTACGTACAAC		Site directed mutagenesis CHIKV nsP2-NTD-Hel-WB
CHIKV-hyb2	AACCCATCATGGATCCTGTGTACGTGGA		In gel hybridization
CHIKV-hyb4	TGTGGGTTCGGAGAATCGTGGAAGAGTT		In gel hybridization
18S	ATGCCCCCGGCCGTCCCTCT		In gel hybridization
VEEV-NTD-Hel-Fw	GCCGAGCTCATGTACCCATACGATGTTCCAGATTACGCTGGCTCAG	SacI + start codon + HA-tag	Amplification VEEV nsP2-NTD-Hel
VEEV-NTD-Hel-Rev	GCCCTCGAGTTAGACGTCGGTAGGGTCCGGTC	Stop codon + XhoI	Prepare cDNA and amplification VEEV nsP2-NTD-Hel

VEEV-QC-WA-Fw	CCAGGATCAGGCGCCTCTGGCATCATTAAAAGC		Site directed mutagenesis VEEV nsP2-NTD-Hel-WA
VEEV-QC-WA-Rev	GCTTTTAATGATGCCAGAGGCGCCTGATCCTGG		Site directed mutagenesis VEEV nsP2-NTD-Hel-WA
VEEV-QC-WB-Fw	CTGTATATTGCCGCCGCTTTTGCTTGTCATGC		Site directed mutagenesis VEEV nsP2-NTD-Hel-WB
VEEV-QC-WB-Rev	GACAAGCAAAAGCGGCGGCAATATACAGGGTC		Site directed mutagenesis VEEV nsP2-NTD-Hel-WB
CHIKV-nsP2-Probe	Texas-AAAACCTGACCCTGGAGACCTCG-BHQ1		qPCR
CHIKV-nsP2-Rev	ACCCTCTGAAGCACGTTAAC		qPCR
CHIKV-nsP2-Fw	AAGCCGATTGTAGTGGACAC		qPCR
Rluc-Probe	FAM-ATCATGGCCTCGTGAAATCCCGT-BHQ1		qPCR
Rluc-Rev	TGTACAACGTCAGGTTTACCAC		qPCR
Rluc-Fw	CAAAGAGAAAGGTGAAGTTCGTC		qPCR
